# Upconversion luminescence and favorable temperature sensing performance of eulytite-type Sr_3_Y(PO_4_)_3_:Yb^3+^/Ln^3+^ phosphors (Ln=Ho, Er, Tm)

**DOI:** 10.1080/14686996.2019.1659090

**Published:** 2019-08-29

**Authors:** Weigang Liu, Xuejiao Wang, Qi Zhu, Xiaodong Li, Xudong Sun, Ji-Guang Li

**Affiliations:** a Key Laboratory for Anisotropy and Texture of Materials (Ministry of Education), Northeastern University, Shenyang, Liaoning, China; b Institute of Ceramics and Powder Metallurgy, School of Materials Science and Engineering, Northeastern University, Shenyang, Liaoning, China; c College of New Energy, Bohai University, Jinzhou, Liaoning, China; d Research Center for Functional Materials, National Institute for Materials Science, Tsukuba, Ibaraki, Japan

**Keywords:** UC luminescence, optical temperature sensing, sensitivity, 204 Optics / Optical applications, 501 Chemical analyses, 505 Optical / Molecular spectroscopy

## Abstract

Phase-pure eulytite-type Sr_3_Y_0.88_(PO_4_)_3_:0.10Yb^3+^,0.02Ln^3+^ upconversion (UC) phosphors (Ln = Ho, Er, Tm) were synthesized *via* gel-combustion and subsequent calcination at 1250°C. Their UC luminescence, temperature-dependent fluorescence intensity ratio of thermally and/or non-thermally coupled energy levels, and performance of optical temperature sensing were systematically investigated. The phosphors typically exhibit green, orange-red and blue luminescence under 978 nm NIR laser excitation for Ln = Er, Ho and Tm, respectively, which were discussed from two- and three-photon processes. The 524 nm green (Er^3+^), 657 nm red (Ho^3+^) and 476 nm blue (Tm^3+^) main emissions were analyzed to have average decay times of ~52 ± 2, 260.6 ± 0.7 and 117 ± 1 μs, respectively. It was shown that (1) the Er^3+^ doped phosphor has a better overall performance of temperature sensing with thermally coupled ^2^H_11/2_ and ^4^S_3/2_ energy levels, whose maximum absolute (*S*_A_) and relative (*S_R_*) sensitivities are ~5.07 × 10^−3^ K^−1^ at 523 K and ~1.16% at 298 K, respectively; (2) the Ho^3+^ doped phosphor shows maximum *S*_A_ and *S_R_* values of ~0.019 K^−1^ (298–573 K) and 0.42% at 573 K for the non-thermally coupled energy pairs of ^5^F_5_/(^5^F_4_,^5^S_2_) and ^5^I_4_/^5^F_5_, respectively; (3) the Tm^3+^ doped phosphor has a maximum *S*_A_ of ~12.74 × 10^−3^ K^−1^ at 573 K for the non-thermally coupled ^3^F_2,3_/^1^G_4_ energy levels and a maximum *S_R_* of ~1.74% K^−1^ at 298 K for the thermally coupled ^3^F_2,3_/^3^H_4_ levels. Advantages of the current phosphors in optical temperature sensing were also revealed by comparing with other typical UC phosphors.

## Graphical abstract

Phase-pure eulytite-type Sr_3_Y_0.88_(PO_4_)_3_:0.10Yb^3+^,0.02Ln^3+^ upconversion (UC) phosphors (Ln=Ho, Er, Tm) were synthesized *via* gel-combustion, whose property and mechanism of UC luminescence and the performance of optical temperature sensing with thermally and/or non-thermally coupled energy levels were thoroughly investigated.

**Figure:**
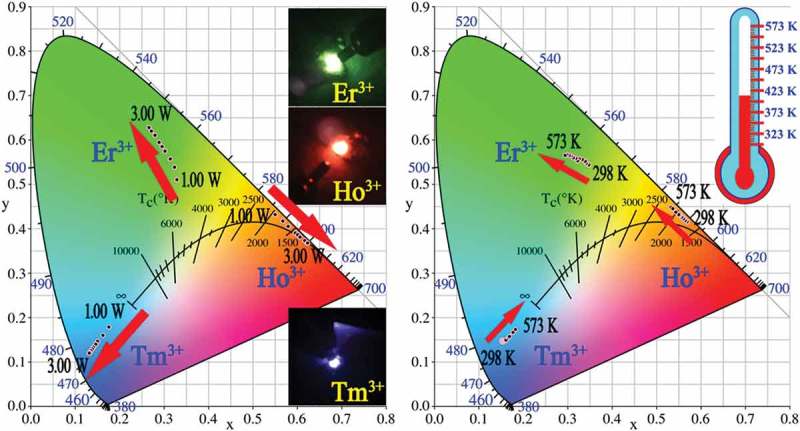

## 1. Introduction

Upconversion (UC) luminescence is a process of converting low-energy light, usually near-infrared (NIR) or infrared, into high-energy light (ultraviolet or visible) through multiple absorption and/or energy transfer [1–3]. UC materials are drawing extensive attention due to their wide applications in the fields of solid-state lasers, multi-color displays, optical communication, wavelength converters for solar cells, bio-imaging, optical temperature sensors, and so forth [1–4]. A UC phosphor is usually formed by doping a host lattice with a sensitizer/activator pair, and the Yb^3+^/Ln^3+^ combination (Ln = Ho, Er, Tm) is the most popular since the ^2^F_7/2_-^2^F_5/2_ transition of Yb^3+^ possesses a large absorption cross-section for ~980 nm NIR excitation light and can well resonate with the ladder-like energy levels of Ho^3+^, Er^3+^ and Tm^3+^ activators [1–3]. The host lattice for UC should assure not only a satisfactory luminescence efficiency, but also excellent physicochemical stability, safety, and low toxicity. A handful of inorganic compounds have been developed as UC host so far, typically including fluorides [5,6], oxides [7,8], oxysulfides [9], phosphates [10,11] and other oxygenates [12–15], and new hosts are also under active exploration and/or perfection.

Optical temperature sensing with UC phosphor, most frequently investigated in the range of ~293–573 K, gained increasing research interest during recent years, which utilizes the fluorescence intensity ratio (FIR) of two emission bands that involve either thermally coupled or non-thermally coupled energy levels of the luminescent ion [4,16]. The FIR technique is usually independent of spectrum loss and excitation-power fluctuation, and may thus provide a high detection resolution and excellent sensitivity [17–19]. The FIR of thermally coupled emission levels is a function of temperature and obeys the Boltzmann distribution of electrons [11]. Since the energy separation Δ*E* of such levels is usually restricted to 200–2000 cm^−1^ to avoid strong overlapping of two emission bands [16–19], the sensitivity of temperature sensing, which is proportional to Δ*E*, can hardly be further improved to a higher level according to the Boltzmann distribution. For this reason, the use of non-thermally coupled energy levels is being considered as an effective complement to a better sensitivity of FIR. Wang et al. [4] have recently reviewed the rare-earth ions, host lattices, electronic transitions and emission wavelengths that have been used for the purpose of optical temperature sensing, together with the temperature ranges of sensing and the absolute/relative sensitivities of FIR.

Eulytite-type orthophosphate M_3_A(PO_4_)_3_ (M = Ca, Sr or Ba; A = La, Gd, Y or Lu) possesses high physical, chemical and structure stabilities [20,21], and may thus serve as an important family of phosphor hosts. It should be noted that many other types of inorganic compounds, such as GdPO_4_ orthophosphate [22] and NaLn(WO_4_)_2_ tungstate (Ln = La-Lu, and Y) [23], also draw great interest for phosphor applications. For downconversion (DC) luminescence, You et al. [24] prepared Eu^2+^/Mn^2+^ co-doped Sr_3_Lu(PO_4_)_3_ by solid-state reaction at 1300°C for 3 h in a CO atmosphere and investigated its luminescence and Eu^2+^-Mn^2+^ energy transfer; Liang et al. [25] produced Ba_3_La(PO_4_)_3_:Ln^3+^ (Ln = Tb, Eu) phosphors via solid reaction at 1200–1250°C for 5–8 h in a thermal carbon atmosphere for color-tunable luminescence; Xia et al. [26] synthesized a series of (Ba,Sr)_3_Lu(PO_4_)_3_:Eu^2+^ phosphors by solid reaction at 1300°C for 4 h under a 10%H_2_-90%N_2_ gas mixture, and the blue shift of Eu^2+^ emission with increasing Sr/Ba ratio was discussed in detail; Xia et al. [27] also synthesized eulytite-type Ba_3_Eu(PO_4_)_3_ and Sr_3_Eu(PO_4_)_3_ compounds via solid reaction at 1250°C for 10 h in air, and systematically compared their crystal structures and photoluminescence; Guo et al. [28] prepared Ba_3_Y(PO_4_)_3_:Eu^2+^/Mn2+ phosphors by solid reaction at 1300°C for 4 h under a 10%H_2_-90%N_2_ gas mixture, and thoroughly investigated their phase formation, luminescence properties and Eu^2+^→Mn^2+^ energy transfer. We have recently synthesized by gel-combustion a series of Ba_3_La(PO_4_)_3_:Ce^3+^/Mn^2+^ and Sr_3_Y(PO_4_)_3_:Eu phosphors [20,21] and evaluated their luminescence. Our thorough literature survey, however, found that the UC luminescence of Yb^3+^/Ln^3+^ pair in eulytite-type M_3_A(PO_4_)_3_ has rarely been investigated up to date, though examples exist for other phosphate hosts such as K_3_Y(PO_4_)_2_ [11] and BiPO_4_ [29]. It is noteworthy that Zhang et al. [14] synthesized in 2018 eulytite-type Ba_3_La(PO_4_)_3_:Yb^3+^/Ln^3+^ phosphors (Ln = Er, Tm) via solid reaction at 1360°C for 5 h in air and studied their properties of temperature sensing with the thermally coupled energy levels of ^2^H_11/2_/^4^S_3/2_ (Er^3+^) and ^3^F_2,3_/^3^H_4_ (Tm^3+^). It was demonstrated that the absolute and relative sensitivities of FIR successively increase (maximum ~4.38 × 10^−3^ K^−1^ at 498 K for Er^3+^ and ~1.31 × 10^−4^ K^−1^ at 503 K for Tm^3+^) and decrease with increasing temperature for both Er^3+^ and Tm^3+^, respectively [14]. While not mentioned for Tm^3+^, a two-photon process was discussed for the UC luminescence of Er^3+^ [14]. The performance of temperature sensing with non-thermally coupled energy levels, however, was not investigated therein for the Ba_3_La(PO_4_)_3_:Yb^3+^/Ln^3+^ phosphors. Compared with Ba_3_La(PO_4_)_3_, the isomorphic Sr_3_Y(PO_4_)_3_ compound can be a better host for UC luminescence, since Y^3+^ is closer to Yb^3+^ and Ln^3+^ (Ln = Ho, Er, Tm) than La^3+^ in ionic radius, which may minimize the lattice distortion upon Yb^3+^/Ln^3+^ doping, and Y is among the most abundant rare-earth elements.

We thus originally synthesized in this work a series of eulytite-type Sr_3_Y(PO_4_)_3_:Yb^3+^/Ln^3+^ UC phosphors (Ln = Ho, Er, Tm) via gel-combustion, followed by a thorough investigation of their UC luminescence and performance of optical temperature sensing with thermally coupled and/or non-thermally coupled energy levels. The high cation homogeneity of sol-gel processing allowed phase-pure products to form by calcination at the lower temperature of 1250°C for 4 h. The current phosphors were also compared with other typical UC systems to show their advantages in optical temperature sensing. In the following sections, we report the synthesis, characterization and optical properties of Sr_3_Y(PO_4_)_3_:Yb^3+^/Ln^3+^ UC phosphors.

## 2. Experimental details

### 2.1. Materials and synthesis

The starting reagents are 99.99% pure RE_2_O_3_ (RE = Y, Ho, Er or Tm; Huizhou Ruier Rare-Chem. Hi-Tech. Co. Ltd., Huizhou, China) and analytical grade ethylenediamine tetraacetic acid (C_10_H_16_N_2_O_8_, EDTA), ammonium hydroxide solution (25 wt%), nitric acid (65 wt%), NH_4_H_2_PO_4_ and Sr(NO_3_)_2_ (Shenyang Chemical Reagent Factory, Shenyang, China). The nitrate solution of RE^3+^ was prepared by dissolving the corresponding oxide with a proper amount of nitric acid, followed by evaporation at 95°C to remove the superfluous HNO_3_ and dilution with distilled water.

For gel-combustion synthesis of Sr_3_Y_0.88_(PO_4_)_3_:0.10Yb^3+^,0.02Ln^3+^, stoichiometric amounts of RE(NO_3_)_3_ and Sr(NO_3_)_2_ were dissolved in an aqueous solution (20 ml) of EDTA-NH_4_OH to chelate the RE^3+^ and Sr^2+^ cations (total cation to EDTA molar ratio = 1:1), followed by the addition of a stoichiometric amount of NH_4_H_2_PO_4_. The mixture was evaporated by heating at 85°C under continuous magnetic stirring to form a sol and then a viscous white gel. Auto-ignition of the gel took place upon increasing the temperature to ~300°C on a resistance oven, which produced a loosely packed black precursor powder. The targeted phosphor was then produced by calcining the precursor in flowing oxygen (200 ml/min) at 1250°C for 4 h [20,21], using a heating rate of 8°C/min at the ramp stage.

### 2.2. Characterization

Phase identification was performed via X-ray diffractometry (XRD, SmartLab, Rigaku, Tokyo, Japan) under 40 kV/200 mA, using nickel-ﬁltered Cu-*K*α radiation (λ = 0.15406 nm) and a scanning speed of 4.0º 2θ per minute. Crystal structure refinement of the product was carried out using the TOPAS 3.0 program [13], and the XRD data for this purpose were acquired in the step scan mode using a step size of 0.02° and an accumulation time of 1.8 s per step. Powder morphology was analyzed via field-emission scanning electron microscopy (FE-SEM, Model S-5000, Hitachi, Tokyo) under an acceleration voltage of 10 kV. UV-Vis spectroscopy was performed at room temperature on a UV-VIS-NIR spectrometer (Model UV-3600 Plus, Shimadzu Co., Kyoto, Japan) equipped with a 150-mm diameter integrating sphere (Model ISR-1503, Shimadzu Co.). UC luminescence of the phosphor was analyzed under 978 nm CW-laser excitation (Model KS3-12322-105, BWT Beijing Ltd., Beijing, China) on an FP-8600 fluorospectrophotometer (JASCO, Tokyo) installed with a heating controller (Model HPC-836, JASCO). Fluorescence decay kinetics was analyzed under 980 nm pulsed laser excitation on a steady-state and transient photoluminescence spectrometer (Model FLS1000, Edinburgh Instruments Ltd., Livingston, UK).

## 3. Results and discussion

### 3.1. Phase analysis and morphology

**Figure 1.**
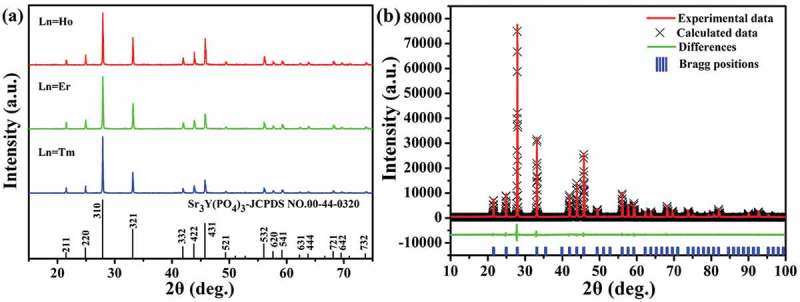
(a) XRD patterns of the as-synthesized Sr_3_Y_0.88_(PO_4_)_3_:0.10Yb^3+^,0.02Ln^3+^ powders, with the standard diffractions of Sr_3_Y(PO_4_)_3_ included as bars for comparison, and (b) Rietveld refinement of the XRD pattern for the Yb^3+^/Er^3+^ doped sample, where the observed, calculated and difference XRD patterns and the positions of Bragg reflections are shown by the red solid line, black crosses, green solid line and blue vertical bars, respectively.

Figure 1(a) presents the powder XRD profiles for the three Sr_3_Y_0.88_(PO_4_)_3_:0.10Yb^3+^,0.02Ln^3+^ phosphors (Ln = Ho, Er or Tm), where it is evident that the patterns are similar to each other and match well with that of cubic structured eulytite-type Sr_3_Y(PO_4_)_3_ in the standard diffraction file (JCPDS No. 00-44-0320; space group: *I*-43*d*). The sharp reflections also indicate that the products were well crystallized. The synthesis temperature is about100°C lower than that (1360°C) needed for the synthesis of Ba_3_La(PO_4_)_3_:Yb^3+^/Ln^3+^ (Ln = Er, Tm) via solid reaction [14], which may originate from the better cation homogeneity of sol-gel processing. The crystal structure of Sr_3_Y(PO_4_)_3_ can be viewed as a three-dimensional connection of [PO_4_]^3-^ tetrahedrons and [(Sr/Y)-O] polyhedrons via corner sharing, where all the [PO_4_]^3-^ are totally independent while the Sr/Y polyhedrons share edges with each other to form a three-dimensional network [26]. In such a structure, the Sr^2+^/Y^3+^ cations are randomly disordered over a single 16c crystallographic site (*C*_3_ point symmetry) [30] but the [PO_4_]^3-^ tetrahedrons show three different orientations in response to three sets of partially occupied oxygen positions O_1_, O_2_ and O_3_ [31]. It is noteworthy that Sr^2+^ and Y^3+^ have different coordination environments although they occupy the same lattice site. Specifically, the Y^3+^ ion resides in YO_6_ octahedron distorted by three equally short and three equally long Y-O bonds [32] while the Sr^2+^ ions have the two coordination environments of CN = 6 and CN = 9 (CN: coordination number) [20]. Accordingly, Sr_3_Y(PO_4_)_3_ presents not only cation disorder but also disorder in the oxygen sublattice [20,32]. In this work, the Yb^3+^ and Ln^3+^ dopants were expected to replace Y^3+^ by valence and size preference (ionic radius *r*= 90.0, 90.1, 89.0, 88.0 and 86.8 pm for Y^3+^, Ho^3+^, Er^3+^, Tm^3+^ and Yb^3+^ under CN = 6; *r*= 118 and 131 pm for Sr^2+^ under CN = 6 and 9, respectively) [33]. Based on this information, Rietveld refinement of the XRD pattern was performed using the standard crystallographic data of isostructural Sr_3_La(PO_4_)_3_ (ICSD No. 69432) as initial structure model. Figure 1(b) shows the experimental and calculated XRD profiles for the Sr_3_Y_0.88_(PO_4_)_3_:0.10Yb^3+^,0.02Er^3+^ representative, while the derived coordinates and site occupancy factors (SOF) of atoms are summarized in Table S1. The refinement was ended up with the well-acceptable reliability factors of *R_wp_*= 8.28%, *R_p_*= 6.12%, *R_exp_*= 3.83% and *χ^2^*= 2.16, and yielded a lattice parameter (*a*= *b*= *c*) of ~10.1043 ± 0.0002 Å and cell volume *V* of ~1031.62 ± 0.05 Å^3^. Similar analysis found the *a* and *V* values of ~10.1045 ± 0.0004 Å and 1031.68 ± 0.12Å^3^ for the Yb^3+^/Ho^3+^ doped and ~10.1024 ± 0.0006 Å and 1031.04 ± 0.18 Å^3^ for the Yb^3+^/Tm^3+^ doped phosphor powders. The cell constants are all smaller than that (10.1091 Å) of Sr_3_Y(PO_4_)_3_ in the standard diffraction file, owing to the smaller average ionic radius of Yb^3+^/Ln^3+^ pair, and tend to decrease toward a smaller Ln^3+^. The above results thus provided persuasive evidence of solid-solution formation.

**Figure 2.**
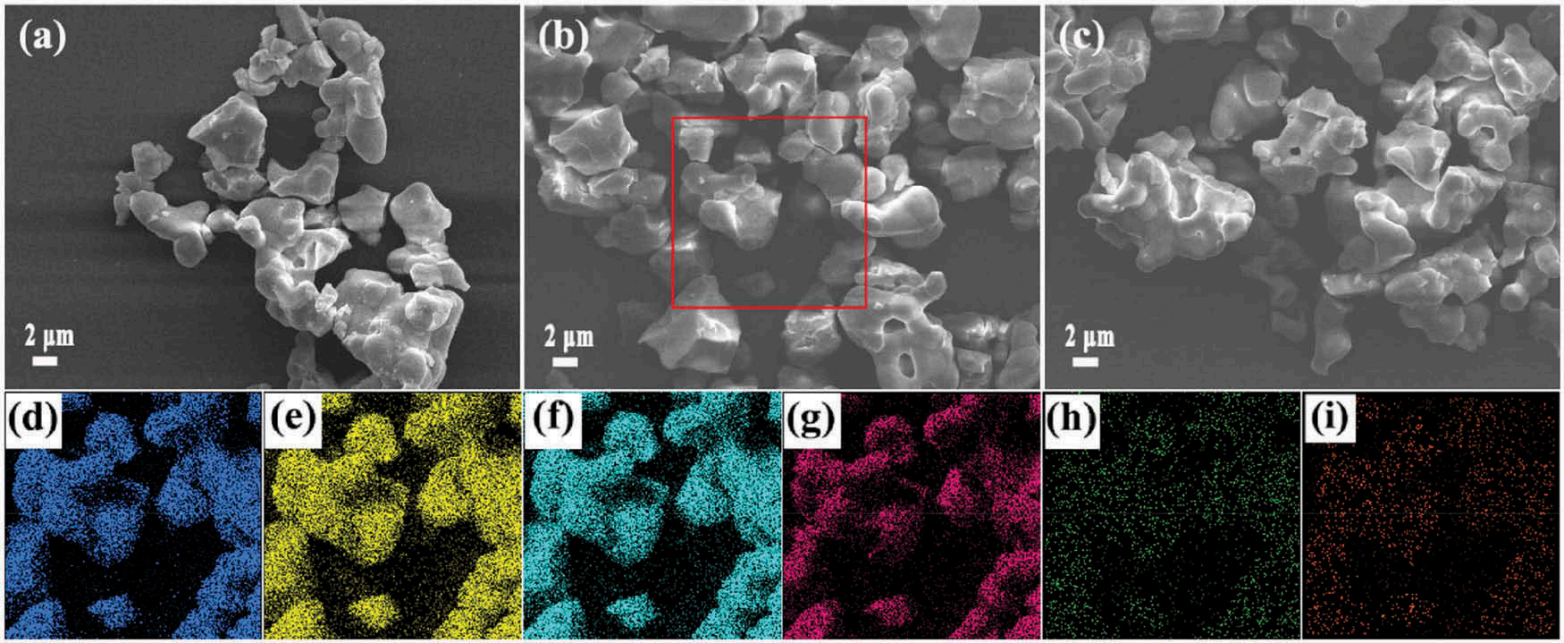
FE-SEM morphologies (upper row) of the Sr_3_Y_0.88_(PO_4_)_3_:0.10Yb^3+^,0.02Ln^3+^ powders, where Ln = Ho (a), Er (b) and Tm (c). The bottom row presents elemental mapping of the red-framed area in (b), with parts (d), (e), (f), (g), (h) and (i) corresponding to elements Sr, Y, P, O, Yb and Er, respectively.

FE-SEM observations (Figure 2(a–c)) reveal that the Sr_3_Y_0.88_(PO_4_)_3_:0.10Yb^3+^,0.02Ln^3+^ products contain aggregated primary particles/crystallites of ~2.0–6.0 μm, which is typical of a gel-combustion product [20,21], and the type of Ln has no appreciable influence on overall morphology of the powder. Elemental mapping via energy dispersive X-ray spectroscopy (EDS), with the Sr_3_Y_0.88_(PO_4_)_3_:0.10Yb^3+^,0.02Er^3+^ sample as a representative, found that all the elements of concern are quite evenly distributed among the particles (Figure 2(d–i)). EDS analysis (Figure S1) also indicated that the sample contains ~15.73 at% of Sr, 4.60 at% of Y, 0.54 at% of Yb, 0.11 at% of Er, 15.81 at% of P and 63.21 at% of O, and the derived Sr:Y:Yb:Er:P:O atomic ratio of 3:0.877:0.103:0.021:3.015:12.055 is very close to the theoretical value of 3:0.88:0.10:0.02:3:12. The above EDS and XRD analyses confirmed that a solid-solution product with the intended chemical composition has been formed.

**Figure 3.**
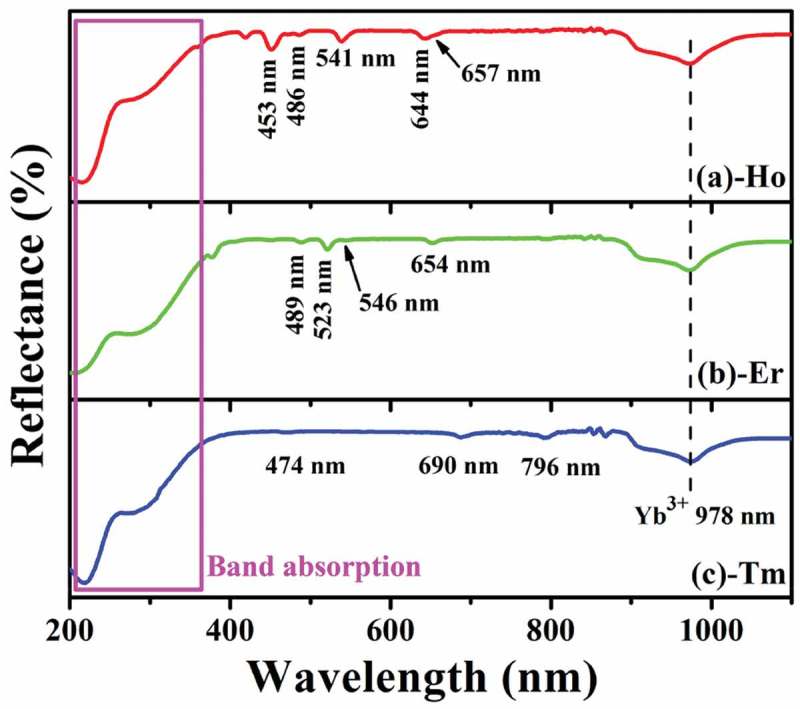
UV-Vis diffuse reflectance spectra of the Sr_3_Y_0.88_(PO_4_)_3_:0.10Yb^3+^,0.02Ln^3+^ powders.

Figure 3 shows the UV-Vis diffuse reflectance spectra of the Sr_3_Y(PO_4_)_3_:Yb^3+^/Ln^3+^ powders, where the broad band in the spectral range of ~200-360 nm and that centered at ~978 nm, which are common to the three samples, can be assigned to absorption by the Sr_3_Y(PO_4_)_3_ host and ^2^F_7/2_→^2^F_5/2_ transition of Yb^3+^, respectively. In addition, the Ho^3 +^ activator clearly shows transitions its ^5^F*_i_*→^5^I_8_ (*i*= 2, 3, 4 and 5) transitions at ~453, 486, 541 and 644/657 nm (Figure 3(a)), Er^3+^ exhibits transitions from the ^4^F_7/2_, ^2^H_11/2_, ^4^S_3/2_ and ^4^F_9/2_ energy states to ^4^I_15/2_ ground state at ~489, 523, 546, 654 nm (Figure 3(b)), and Tm^3+^ shows transitions from the ^1^G_4_, ^3^F_2,3_ and ^3^H_4_ levels to ^3^H_6_ ground state at ~474, 690 and 796 nm (Figure 3(c)), respectively. The results thus imply that the Sr_3_Y(PO_4_)_3_:Yb^3+^/Ln^3+^ powders can effectively absorb 978 nm laser excitation for UC luminescence. The energy bandgap of Sr_3_Y(PO_4_)_3_:Yb^3+^/Ln^3+^ can be estimated from the reflectance spectra according to Equation (1) [34,35]

(1) $$[F(R_{\infty })h\nu {]}^{n}=A(h\nu -E_{g})$$

where *hv* is the incident photon energy, *A* is a proportional constant, *E*_g_ is the value of bandgap, *n*= 2 for a direct transition or 1/2 for an indirect transition, and F(*R*_∞_) is the Kubelka-Munk function which is defined as [36,37]

(2) $$F(R_{\infty })=(1-R{)}^{2}/2R=K/S$$

where *R, K* and *S* are the reflection, absorption and scattering coefficients, respectively. The [F(*R*_∞_)*hv*]^1/2^ *vs hv* plots are shown in Figure S2, where extrapolating the linear portions to [F(*R*_∞_)*hv*]^1/2^ = 0 yielded the similar *E*_g_ values of ~3.37 eV. The *E*_g_ value is also close to those reported for the isostructural Ba_3_La(PO_4_)_3_ (3.46 eV) [38], Ba_3_Y(PO_4_)_3_ (3.15 eV) [39] and Sr_3_Gd(PO_4_)_3_ (3.49 eV) [40] compounds synthesized by solid reaction.

### 3.2. Upconversion luminescence of the Sr_3_Y(PO_4_)_3_:Yb^3+^/Ln^3+^ phosphors

**Figure 4.**
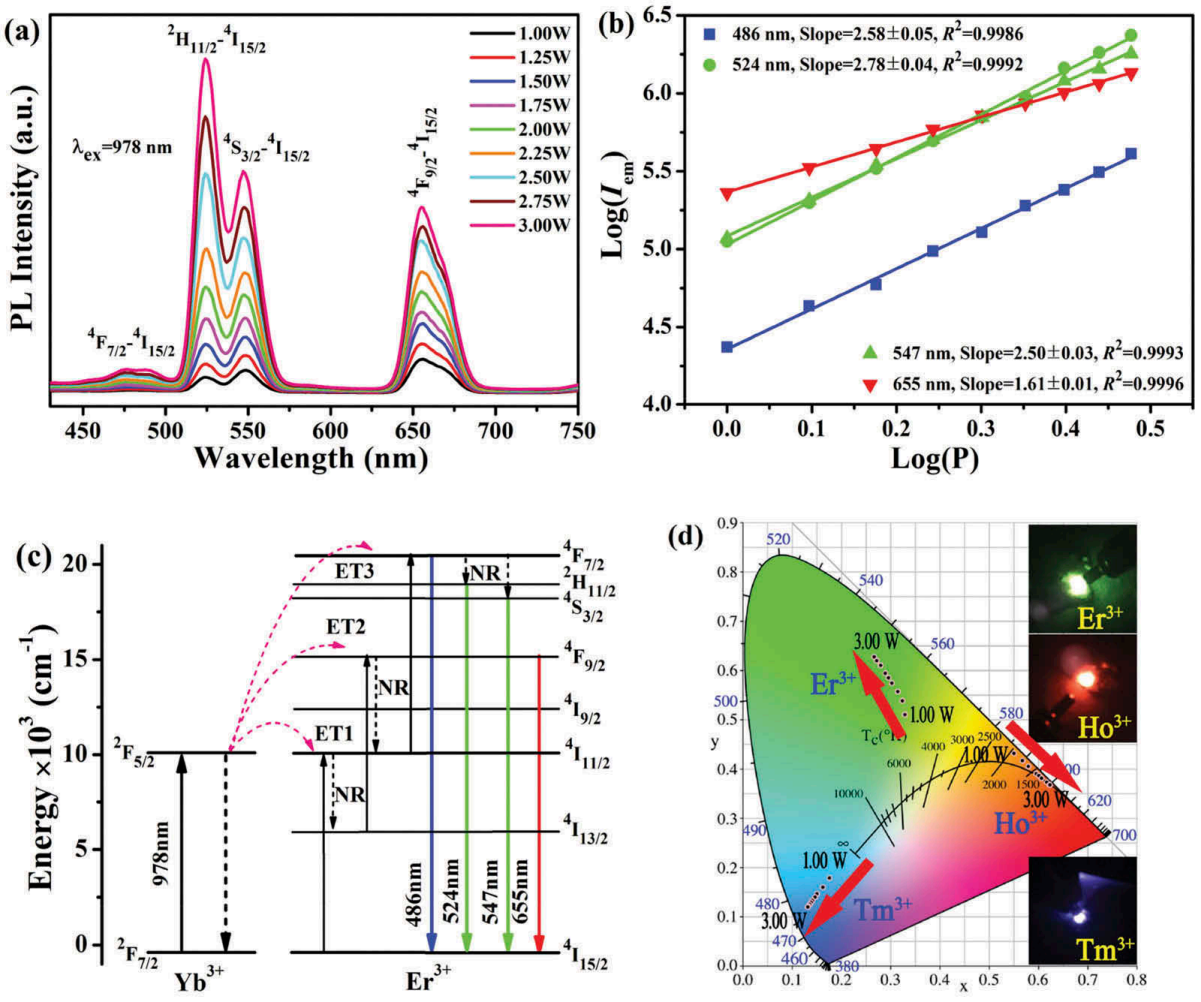
UC luminescence spectra under different excitation power levels (a), the relationship between log(*I*_em_) and log*P* (in Watt, b), and a scheme showing the energy levels and UC process (c) for the Sr_3_Y_0.88_(PO_4_)_3_:0.10Yb^3+^,0.02Er^3+^ phosphor. Part (d) shows the CIE chromaticity coordinates of Sr_3_Y_0.88_(PO_4_)_3_:0.10Yb^3+^,0.02Ln^3+^ under varying excitation power, where the inset photographs are for the appearances of UC luminescence under 2.00 W of 978 nm laser excitation.

Yb^3+^/Er^3+^ is the most widely investigated sensitizer/activator pair for UC luminescence in various types of host lattices [9–19], since the ^2^F_5/2_→^2^F_7/2_ emission of Yb^3+^ and the ^4^I_15/2_→^4^I_11/2_ excitation transition of Er^3+^ have well-matching energies. Figure 4(a) shows the UC luminescence spectra of Sr_3_Y_0.88_(PO_4_)_3_:0.10Yb^3+^,0.02Er^3+^ under varying pumping power of 978 nm laser. It is seen that, in each case, the spectrum includes a blue (~486 nm, negligibly weak), green (~524/547 nm, dominantly strong) and red (~655 nm, strong) band in the visible-light region, which are assignable to transitions from the ^4^F_7/2_, ^2^H_11/2_/^4^S_3/2_ and ^4^F_9/2_ excited states to the^4^I_15/2_ ground state of Er^3+^ [41,42], respectively. Increasing power of excitation did not bring about any change to peak position but monotonically raised the emission intensity of each band. The Commission International de L’Eclairage (CIE) chromaticity coordinates of UC luminescence are summarized in Figure 4(d) and Table S2, where it is clear that the emission color steadily drifted from yellowish green [color coordinates: (0.3288, 0.5102)] to green [color coordinates: (0.2671, 0.6276)] with increasing excitation power from 1.00 to 3.00 W. The color change agrees with the almost linearly increasing intensity ratio of green to red emission (*I*_524_/*I*_655_ and *I*_547_/*I*_655_, Figure S3(a)). Under 2.00 W laser pumping, vivid and strong green emission was observed for Sr_3_Y_0.88_(PO_4_)_3_:0.10Yb^3+^,0.02Er^3+^ with naked eyes, as shown by the inset photograph taken for the appearance of luminescence in Figure 4(d).

The number of excitation photons required to populate the upper emitting state under unsaturated condition can be obtained from the relation *I*_em_∝*P^n^* [9,13], where *I*_em_ is the emission intensity, *P* is the pumping power, and *n* is the number of low-energy photons required to convert to one high-energy photon in the UC process. Figure 4(b) shows the log(*I*_em_)-log(*P*) plot of the above relation, from which the *n* values were determined from the slope of the linear fitting to be ~2.58, 2.78, 2.50 and 1.61 for the UC peaks at ~486, 524, 547 and 655 nm, respectively. The results thus suggest that a three-photon process is primarily responsible for the observed ^4^F_7/2_→^4^I_15/2_ (486 nm), ^2^H_11/2_→^4^I_15/2_ (524 nm) and ^4^S_3/2_→^4^I_15/2_ (547 nm) emissions and a two-photon process for the ^4^F_9/2_→^4^I_15/2_ (655 nm) one. The process of UC is known to be virtually affected a number of factors, such as host composition, lattice defects, crystallinity, the content, distribution uniformity and actual lattice site of the sensitizer/activator pair, and so forth [1–4,12]. The results of this work are consistent with those obtained from YbPO_4_:Er [10] and NaLu(WO_4_)_2_:Yb/Er [12] UC phosphors, though a three-photon process was reported for all the ^2^H_11/2_/^4^S_3/2_→^4^I_15/2_ and ^4^F_9/2_→^4^I_15/2_ emissions of La_2_O_2_SO_4_:Yb/Er [13] and a two-photon mechanism for each emission of La_2_O_2_S:Yb/Er [9], KMgF_3_:Yb/Er [43], Ba_5_Gd_8_Zn_4_O_21_:Yb/Er [44], α-NaYF_4_:Yb/Er [45] and Na_2_Y_2_B_2_O_7_:Yb/Er [46]. The three basic mechanisms of excited state absorption (ESA), energy transfer (ET) and photon avalanche (PA) have been proposed for UC luminescence [1–3]. Since no power threshold was observed in the range of this study, the avalanche mechanism can be neglected. With the energy diagram constructed in Figure 4(c) by referring to previous studies, the UC luminescence of Sr_3_Y_0.88_(PO_4_)_3_:0.10Yb^3+^,0.02Er^3+^ was proposed to occur via the following processes:

(1) The Yb^3+^ electrons are excited from the ^2^F_7/2_ ground state to the ^2^F_5/2_ level by absorbing the energy of one laser photon [ESA, ^2^F_7/2_ (Yb^3+^) + *hν* (978 nm) → ^2^F_5/2_ (Yb^3+^)], which transfer energy to Er^3+^ while returning to the ^2^F_7/2_ ground state and thus promotes Er^3+^ electrons from the ^4^I_15/2_ ground state to the ^4^I_11/2_ level [ET1, ^2^F_5/2_ (Yb^3+^) + ^4^I_15/2_ (Er^3+^) → ^2^F_7/2_ (Yb^3+^) + ^4^I_11/2_ (Er^3+^)]; (2) After nonradiative relaxation to ^4^I_13/2_ [NR, ^4^I_11/2_ (Er^3+^) ~ ^4^I_13/2_ (Er^3+^)], Er^3+^ electrons are raised to the ^4^F_9/2_ level via energy transfer of a second laser photon [ET2, ^2^F_5/2_ (Yb^3+^) + ^4^I_13/2_ (Er^3+^) → ^2^F_7/2_ (Yb^3+^) + ^4^F_9/2_ (Er^3+^)]; (3) A part of the ^4^F_9/2_ electrons radiatively relax to the ^4^I_15/2_ ground state, which produces the ~655 nm red emission (^4^F_9/2_→^4^I_15/2_), and the other part relax to the ^4^I_11/2_ state via NR [^4^I_9/2_ (Er^3+^) ~ ^4^I_11/2_ (Er^3+^)], followed by further excitation to the ^4^F_7/2_ level with a third laser photon [ET3, ^2^F_5/2_ (Yb^3+^) + ^4^I_11/2_ (Er^3+^) → ^2^F_7/2_ (Yb^3+^) + ^4^F_7/2_ (Er^3+^)]. The ^4^F_7/2_ electrons may directly jump back to the ^4^I_15/2_ ground state to produce the ~486 nm blue emission (^4^F_7/2_→^4^I_15/2_) and may relax to the ^2^H_11/2_/^4^S_3/2_ levels via NR, from which the ~524/547 nm green emissions can be resulted upon back-jumping of the electrons to the ^4^I_15/2_ ground level (^2^H_11/2_/^4^S_3/2_→^4^I_15/2_). The very weak ^4^F_7/2_→^4^I_15/2_ blue emission suggests that most of the excitation energy accumulated by ET3 relaxes to the ^2^H_11/2_/^4^S_3/2_ states to result in the strong green emission (Figure 4(a)). The gradually larger green to red intensity ratio (Figure S3(a)) may imply that the ^2^H_11/2_/^4^S_3/2_ levels gain population faster than ^4^F_9/2_ under a higher excitation power.

**Figure 5.**
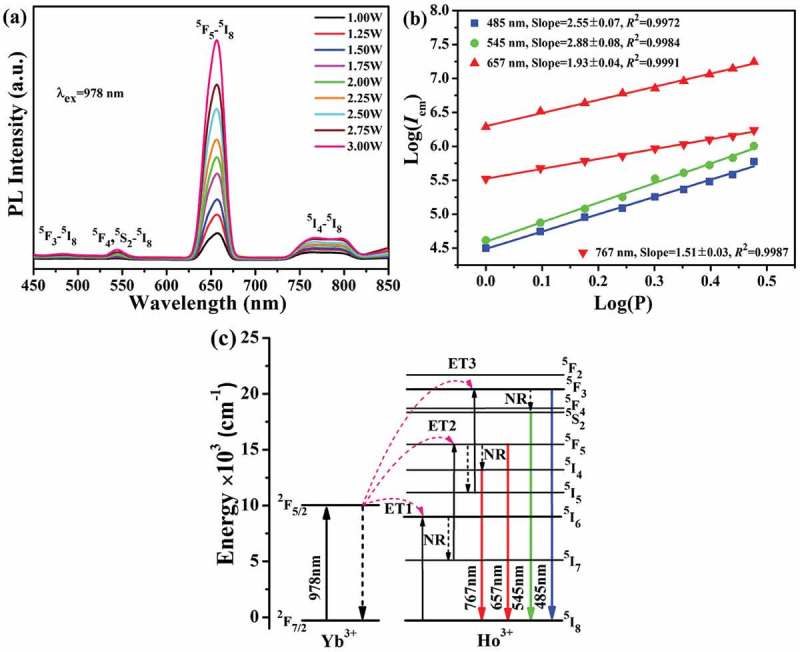
UC luminescence spectra under different excitation power levels (a), the relationship between log(*I*_em_) and log*P* (in Watt, b), and a scheme showing the energy levels and UC process (c) for the Sr_3_Y_0.88_(PO_4_)_3_:0.10Yb^3+^,0.02Ho^3+^ phosphor.

Figure 5(a) shows the UC luminescence spectra of Yb^3+^/Ho^3+^ codoped Sr_3_Y(PO_4_)_3_ under varying excitation power, where the four groups of emission bands centered at ~485 nm (blue, negligible), 545 nm (green, weak), 657 nm (red, overwhelmingly strong) and 767 nm (red in NIR, weak) can be assigned to ^5^F_3_→^5^I_8_, ^5^F_4_/^5^S_2_→^5^I_8_, ^5^F_5_→^5^I_8_ and ^5^I_4_→^5^I_8_ transitions of Ho^3+^ [9–12], respectively. Raising excitation power from 1.00 to 3.00 W did not produce any new emission but successively improved the intensity of the existing luminescence. The CIE chromaticity coordinates of UC luminescence gradually drifted from orange [(0.5503,0.4318)] to orange-red [(0.6235,0.3676)] with increasing excitation power (Figure 4(d) and Table S2), which is due to the gradually larger red to green intensity ratio (*I*_657_/*I*_545_, Figure S3(b)). Under 2.00 W laser pumping, the phosphor exhibits a vivid and strong orange-red emission visible to naked eyes, as shown by the inset in Figure 4(d).

Analysis of the log(*I*_em_)-log(*P*) plots found *n* values of ~2.55, 2.88, 1.93 and 1.51 for the ~485, 545, 657 and 767 nm UC bands (Figure 5(b)), respectively, which indicate that a three-photon process is largely responsible for the ^5^F_3_→^5^I_8_ (485 nm) and ^5^F_4_/^5^S_2_→^5^I_8_ (545 nm) transitions while a two-photon process for the ^5^F_5_→^5^I_8_ (657 nm) and ^5^I_4_→^5^I_8_ (767 nm) transitions of Ho^3+^. The UC luminescence of Y_2_O_3_:Yb^3+^/Ho^3+^/Zn^2+^(YOZ) [8] and BaZrO_3_:Yb^3+^/Ho^3+^(BZ) [47] were also reported to involve three- (^5^F_3_→^5^I_8_ of YOZ; ^5^F_4_/^5^S_2_→^5^I_8_ of BZ) and two- (^5^F_4_/^5^S_2_→^5^I_8_ and ^5^F_5_→^5^I_8_ of YOZ; ^5^F_5_→^5^I_8_ of BZ) photon processes, although a three-photon process was suggested for all the emissions of La_2_O_2_S:Yb/Ho [9], YbPO_4_:Ho [10], NaLu(WO_4_)_2_:Yb/Ho [12] and La_2_O_2_SO_4_:Yb/Ho [13] and a two-photon process for those of Sr_5_(PO_4_)_3_Cl:Yb/Ho [48]. With the energy level diagram constructed in Figure 5(c), the UC process of Sr_3_Y_0.88_(PO_4_)_3_:0.10Yb^3+^,0.02Ho^3+^ can be described with the following photon reactions:

(1) ESA: ^2^F_7/2_ (Yb^3+^) + *hν* (978 nm) → ^2^F_5/2_ (Yb^3+^);

(2) ET1: ^2^F_5/2_ (Yb^3+^) + ^5^I_8_ (Ho^3+^) → ^2^F_7/2_ (Yb^3+^) + ^5^I_6_ (Ho^3+^);

(3) NR: ^5^I_6_ (Ho^3+^) ~ ^5^I_7_ (Ho^3+^);

(4) ET2: ^2^F_5/2_ (Yb^3+^) + ^5^I_7_ (Ho^3+^) → ^2^F_7/2_ (Yb^3+^) + ^5^F_5_ (Ho^3+^);

(5) Emission: ^5^F_5_ (Ho^3+^) → ^5^I_8_ (Ho^3+^) + *hν* (657 nm);

(6) NR: ^5^F_5_ (Ho^3+^) ~ ^5^I_4_ (Ho^3+^);

(7) Emission: ^5^I_4_ (Ho^3+^) → ^5^I_8_ (Ho^3+^) + *hν* (767 nm);

(8) NR: ^5^F_5_ (Ho^3+^) ~ ^5^I_5_ (Ho^3+^);

(9) ET3: ^2^F_5/2_ (Yb^3+^) + ^5^I_5_ (Ho^3+^) → ^2^F_7/2_ (Yb^3+^) + ^5^F_3_ (Ho^3+^);

(10) Emission: ^5^F_3_ (Ho^3+^) → ^5^I_8_ (Ho^3+^) + *hν* (485 nm);

(11) NR: ^5^F_3_ (Ho^3+^) ~ ^5^F_4_/^5^S_2_ (Ho^3+^);

(12) Emission: ^5^F_4_/^5^S_2_ (Ho^3+^) → ^5^I_8_ (Ho^3+^) + *hν* (545 nm).

The overwhelmingly strong red emission (~657 nm) may have two origins: (1) most of the ^5^F_5_ electrons excited by ET2 directly transit back to the ^5^I_8_ ground state (the *n*= 2 channels), and (2) the ^5^F_3_ electrons excited by ET3 decay to the ^5^F_5_ level, followed by radiative transition to the ^5^I_8_ state (the *n*= 3 channel). The linearly increasing *I*_657_/*I*_485_ and *I*_657_/*I*_545_ intensity ratios (Figure S3(b)) may imply that a higher excitation power leads to a faster population of the ^5^F_5_ energy state.

**Figure 6.**
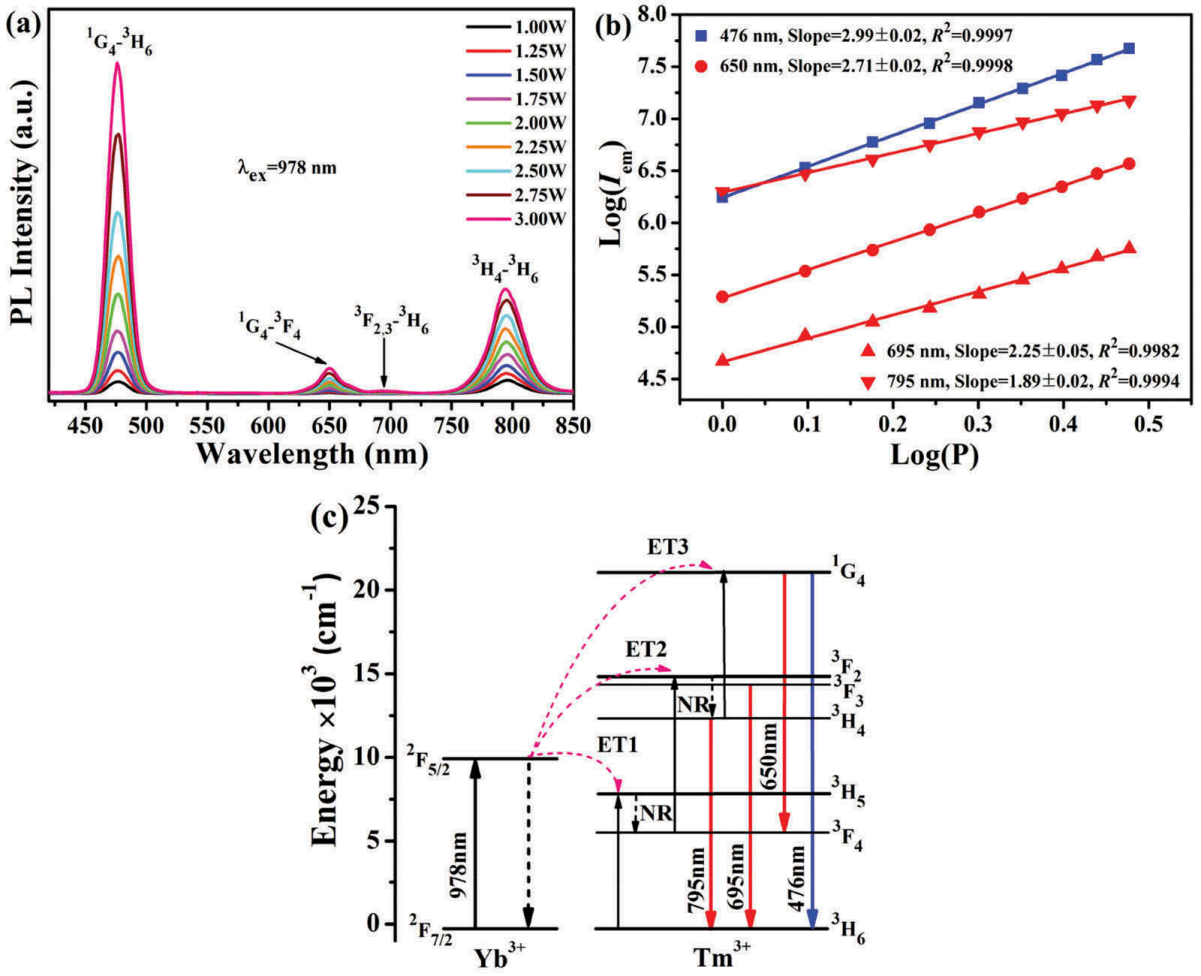
UC luminescence spectra under different excitation power levels (a), the relationship between log(*I*_em_) and log*P* (in Watt, b), and a scheme showing the energy levels and UC process (c) for the Sr_3_Y_0.88_(PO_4_)_3_:0.10Yb^3+^,0.02Tm^3+^ phosphor.

Under 978 nm laser excitation, Sr_3_Y_0.88_(PO_4_)_3_:0.10Yb^3+^, 0.02Tm^3+^ phosphor exhibits four groups of emissions at ~476 nm (blue), ~650 nm (red), 695 nm (red, negligible) and ~795 nm (NIR) as shown in Figure 6(a), which correspond to the ^1^G_4_→^3^H_6_, ^1^G_4_→^3^F_4_, ^3^F_2,3_ →^3^H_6_ and ^3^H_4_→^3^H_6_ transitions of Tm^3+^, respectively. Increasing excitation power led to faster enhancement of blue emission, which became dominant when *P* reached ~1.50 W. The strong blue emission is evident from the appearance of UC luminescence under 2.00 W of laser pumping (Figure 4(d), the inset). It is also seen from Figure 4(d) and Table S2 that the emission color gradually drifted from light blue [(0.1764, 0.1781)] to deep blue [(0.1323, 0.1194)] with increasing excitation power from 1.00 to 3.00 W, which conforms to the gradually larger blue to red intensity ratio (*I*_476_/*I*_650_, Figure S3(c)). Analyzing the emission intensity against excitation power yielded slope (*n*) values of ~2.99 and 2.71 (around 3) for the ~476 nm (^1^G_4_→^3^H_6_) and 650 nm (^1^G_4_→^3^F_4_) UC bands and ~2.25 and 1.89 (around 2) for the ~695 nm (^3^F_2,3_ →^3^H_6_) and 795 nm (^3^H_4_→^3^H_6_) ones (Figure 6(b)), which correspond to three- and two-photon UC mechanisms, respectively. Similar results were reported in the literature for *β*-NaLuF_4_:Yb/Tm [10], Ba_5_Gd_8_Zn_4_O_21_:Yb/Tm [49] and LiLa(MoO_4_)_2_:Yb/Tm [50], though a three-photon process was proposed for the UC emissions of NaLu(WO_4_)_2_:Yb/Tm [12], La_2_O_2_SO_4_:Yb/Tm [13] and La_2_O_2_S:Yb/Tm [51]. By referring to the energy level diagram shown in Figure 6(c), the processes that led to the observed UC luminescence may be presented as follows:

(1) ESA: [^2^F_7/2_ (Yb^3+^) + *hν* (978 nm) → ^2^F_5/2_ (Yb^3+^)];

(2) ET1: [^2^F_5/2_ (Yb^3+^) + ^3^H_6_ (Tm^3+^) → ^2^F_7/2_ (Yb^3+^) + ^3^H_5_ (Tm^3+^)];

(3) NR: [^3^H_5_ (Tm^3+^) ~ ^3^F_4_ (Tm^3+^)];

(4) ET2: [^2^F_5/2_ (Yb^3+^) + ^3^F_4_ (Tm^3+^) → ^2^F_7/2_ (Yb^3+^) + ^3^F_2,3_ (Tm^3+^)];

(5) Emission: ^3^F_2,3_ (Tm^3+^) → ^3^H_6_ (Tm^3+^) + *hν* (695 nm);

(6) NR: ^3^F_2,3_ (Tm^3+^) ~ ^3^H_4_ (Tm^3+^)];

(7) Emission: ^3^H_4_ (Tm^3+^) → ^3^H_6_ (Tm^3+^) + *hν* (795 nm);

(7) ET3: ^2^F_5/2_ (Yb^3+^) +^3^H_4_ (Tm^3+^) → ^2^F_7/2_ (Yb^3+^) + ^1^G_4_ (Tm^3+^);

(8) Emission: ^1^G_4_ (Tm^3+^) → ^3^H_6_ (Tm^3+^) +*hν* (476 nm) and ^1^G_4_ (Tm^3+^) → ^3^F_4_ (Tm^3+^) + *hν* (650nm).

The negligibly weak 695 nm red emission indicates that only a very limited number of the electrons excited by ET2 to the ^3^F_2,3_ level directly decay to the ^3^H_6_ ground state. On the other hand, the much faster intensity increase of 476 nm blue emission suggests a preferential population of the ^1^G_4_ energy level under increasing excitation power.

**Figure 7.**
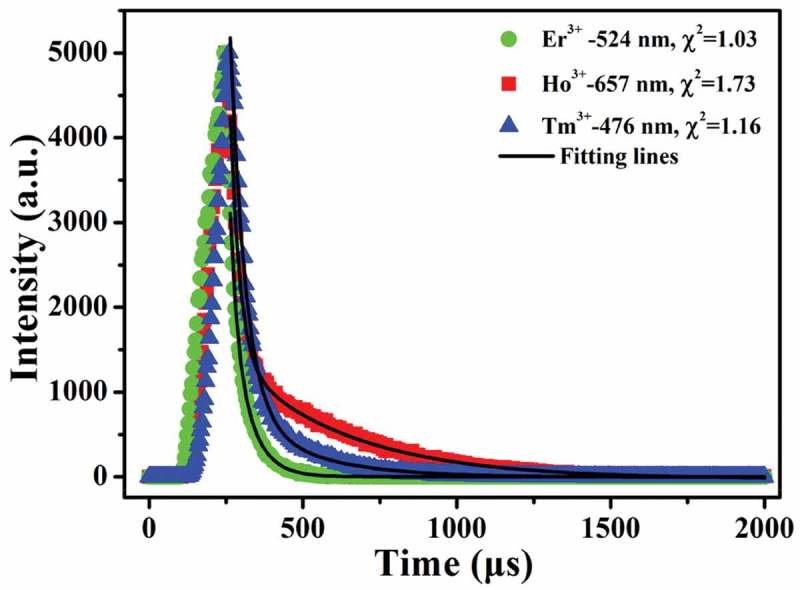
Temporal evolution of the 524 nm green, 657 nm red and 476 nm blue emissions of Er^3+^, Ho^3+^ and Tm^3+^, respectively.

Figure 7 exhibits fluorescence decay curves for the green emission of Er^3+^ (524 nm, ^2^H_11/2_→^4^I_15/2_ transition), red emission of Ho^3+^ (657 nm, ^5^F_5_→^4^I_8_ transition) and blue emission of Tm^3+^ (476 nm, ^1^G_4_→^3^H_6_). It was found that the decay curve can be well fitted with the second-order exponential equation *I*(*t*) = *A*_1_exp(-t/*τ*_1_)+*A*_2_exp(-t/*τ*_2_)+*B* in each case, where *I*(t) is the fluorescence intensity at time t, *A*_1_ and *A*_2_ are pre-exponential constants, τ_1_ and τ_2_ stand for the decay time of exponential components, and *B* is a constant. The average lifetime (τ*) can be calculated with the following formula [20,41]:

(3) $$\tau *=\frac{A_{1}\tau_{1}^{2}+A_{2}\tau_{2}^{2}}{A_{1}\tau_{1}+A_{2}\tau_{2}}$$

The derived *τ*_1_ and *τ*_2_ values and their weights are tabulated in Table S3, from which τ* values of ~52 ± 2, 260.6 ± 0.7 and 117 ± 1 μs were obtained for the aforesaid emissions of Er^3+^, Ho^3+^ and Tm^3+^, respectively. The short fluorescence lifetime would be beneficial to temporal and spatial resolution of temperature measurement.

**Figure 8.**
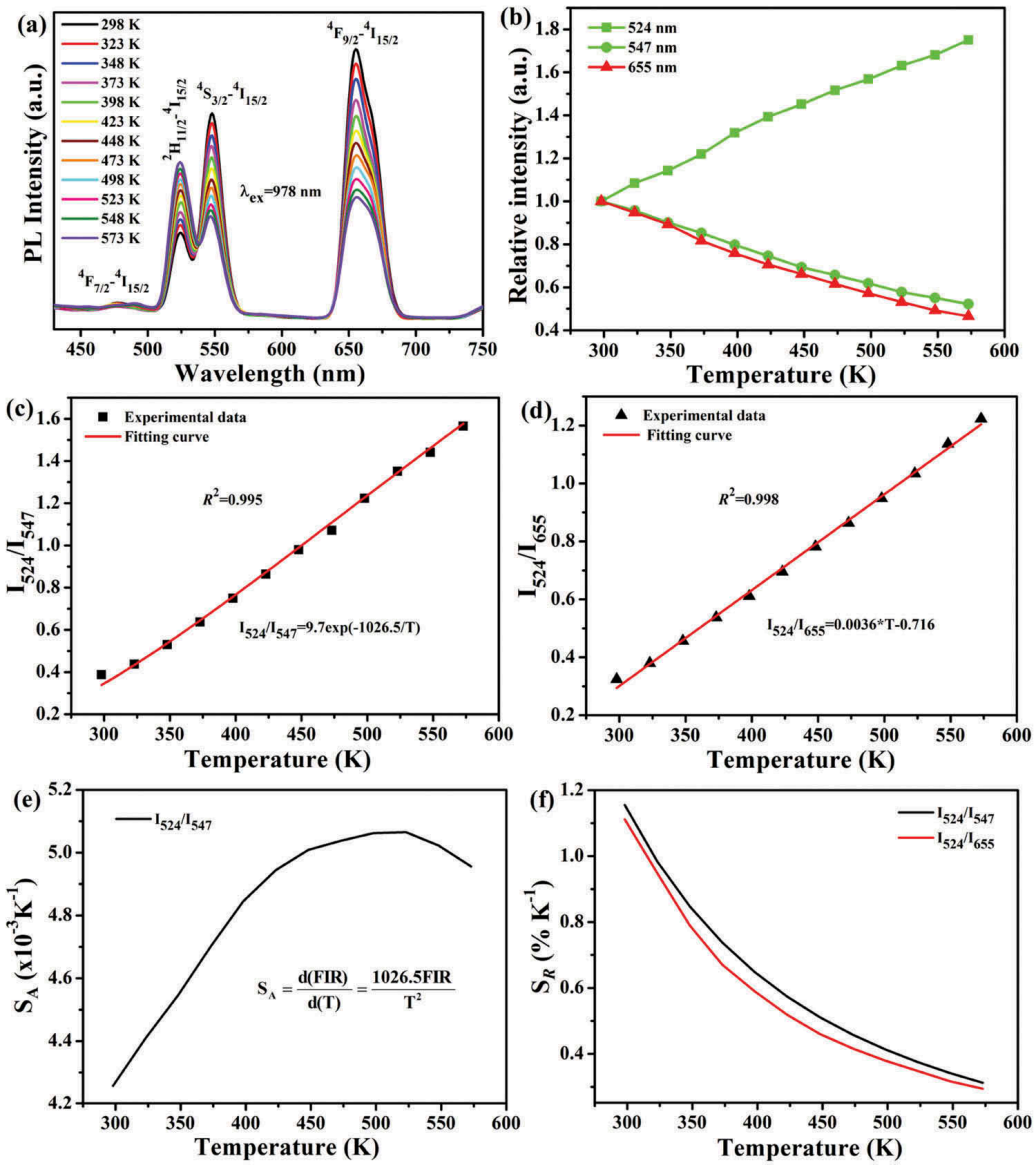
Temperature-dependent emission spectra under 1.00 W of 978 nm laser excitation (a), relative intensities of the 524, 547 and 655 nm emissions as a function of measurement temperature (b), the dependences of I_524_/I_547_ (c) and I_524_/I_655_ (d) FIRs on the absolute temperature, the absolute sensitivity of I_524_/I_547_ FIR (e) and the relative sensitivities of I_524_/I_547_ and I_524_/I_655_ FIRs (f) of the Sr_3_Y_0.88_(PO_4_)_3_:0.10Yb^3+^,0.02Er^3+^ phosphor.

### 3.3. Temperature sensing performance of Sr_3_Y(PO_4_)_3_:Yb^3+^/Ln^3+^ UC phosphors

Figure 8(a,b) present the temperature-dependent UC spectra and relative emission intensity of Sr_3_Y_0.88_(PO_4_)_3_:0.10Yb^3+^,0.02Er^3+^ phosphor under 1.00 W of 978 nm laser excitation, respectively. It is clear that the 524 nm green emission (^2^H_11/2_→^4^I_15/2_) gains intensity while the 547 nm green (^4^S_3/2_→^4^I_15/2_) and 655 nm red (^4^F_9/2_→^4^I_15/2_) emissions lose intensity with increasing temperature. The opposite trends observed for the two green bands can be ascribed to thermal coupling of the ^2^H_11/2_ and ^4^S_3/2_ levels [16–18], while intensity loss of the red emission is mostly due to enhanced non-radiative relaxation from the ^4^F_9/2_ level by intensified lattice vibration at a higher temperature.^16^ The fluorescence intensity ratio (FIR) of thermally coupled ^2^H_11/2_/^4^S_3/2_ levels follows Boltzmann distribution, and can be described as [11,16–19,44,52]

(4) $$FIR=\frac{I_{524}}{I_{547}}=N\exp(\frac{-\Delta E}{kT})$$

where Δ*E* is the energy gap between the ^2^H_11/2_ and ^4^S_3/2_ levels, *k* is the Boltzmann constant (0.695 K^−1^cm^−1^), *T* is the absolute temperature, and *N* is a proportionality constant. Figure 8(c) shows the temperature dependence of *I*_524_/*I*_547_ FIR, where it was found that the experimental data can be well fitted with the single-exponential equation of FIR(*I*_524_/*I*_547_) = 9.7exp(−1026.5/*T*). The derived Δ*E* of ~713 ± 4 cm^−1^ agrees with the energy gap (700–800 cm^−1^) between ^2^H_11/2_ and ^4^S_3/2_ [11]. Figure 8(d) shows *I*_524_/*I*_655_ FIR for the non-thermally coupled levels of ^2^H_11/2_ and ^4^F_9/2_, where it was found that the experimental data can be satisfactorily fitted with the linear equation of FIR(*I*_524_/*I*_655_) = 0.0036*T-0.716.

Absolute (*S*_A_) and relative (*S_R_*) sensitivities are two indispensable parameters for temperature sensing, which can be calculated using the following formulas [14,16–18,53]:

(5) $$S_{A}=\left|\frac{d(FIR)}{d(T)}\right|=FIR*(\frac{\Delta E}{kT^{2}})$$

(6) $$S_{R}=\left|\frac{1}{FIR}\frac{d(FIR)}{d(T)}\right|=\frac{\Delta E}{kT^{2}}$$

For the thermally coupled ^2^H_11/2_/^4^S_3/2_ levels, it was observed that the *S*_A_ of *I*_524_/*I*_547_ FIR first increases to reach its maximum of ~5.07 × 10^−3^ K^−1^ at 523 K and then slightly decreases (Figure 8(e)). The non-thermally coupled ^2^H_11/2_ and ^4^F_9/2_ levels have an *S*_A_ of ~3.6 × 10^−3^ K^−1^ for *I*_524_/*I*_655_ FIR, according to the linear fitting in Figure 8(d), which is generally smaller than the *S*_A_ of *I*_524_/*I*_547_ FIR (Figure 8(e)). As compared in Table 1, our phosphor has a significantly better absolute sensitivity (*S*_A_) than Yb^3+^/Er^3+^ codoped Y_2_O_3_ [7], K_3_Y(PO_4_)_2_ [11], Ca_3_La_6_Si_6_O_24_ [18], NaYF_4_ [52], and Ba_3_La(PO_4_)_3_ [14].

**Table 1.** A summary of *S*_A_ and *S_R_* values, electronic transitions and temperature sensing ranges for some typical temperature sensing UC phosphors doped with Yb^3+^/Ln^3+^ pair.

Ln^3+^	Host	Transition/wavelength (nm)	Range (K)	S_A_ (K^−1^) (maximum)	S*_R_* (K^−1^)	Ref.
Er^3+^	Y_2_O_3_	^2^H_11/2_/^4^S_3/2_→^4^I_15/2_(525/550)	93–613	4.4 × 10^−3^(427K)	886.08/*T*^2^	[7]
Er^3+^	K_3_Y(PO_4_)_2_	^2^H_11/2_/^4^S_3/2_→^4^I_15/2_(520/555)	293–553	3.04 × 10^−3^(553K)	1127.5/*T*^2^	[11]
Er^3+^	Ba_3_La(PO_4_)_3_	^2^H_11/2_/^4^S_3/2_→^4^I_15/2_(522/545)	298–498	4.38 × 10^−3^(498K)	1002/*T*^2^	[14]
Er^3+^	Ca_3_La_6_Si_6_O_24_	^2^H_11/2_/^4^S_3/2_→^4^I_15/2_(522/548)	293–573	3.91 × 10^−3^(500K)	1008/*T*^2^	[18]
Er^3+^	*α*-NaYF_4_	^2^H_11/2_/^4^S_3/2_→^4^I_15/2_(524/545)	303–573	4.54 × 10^−3^(541K)	1085.3/*T*^2^	[52]
Er^3+^	*β*-NaYF_4_	^2^H_11/2_/^4^S_3/2_→^4^I_15/2_(524/545)	303–573	4.84 × 10^−3^(515K)	1025.8/*T*^2^	[52]
Ho^3+^	Y_2_O_3_	^5^F_3_/^3^K_8_ →^5^I_8_(465/491)	299–673	3.02 × 10^−3^ (673 K)	1067.76/*T*^2^	[8]
Ho^3+^	K_3_Y(PO_4_)_2_	^5^F_5_/(^5^F_4_,^5^S_2_)→^5^I_8_(659/545)	303–523	0.078(303-523K)	0.20%(303K)	[11]
Ho^3+^	BaY_2_Si_3_O_10_	^5^F_5_/(^5^F_4_,^5^S_2_)→^5^I_8_(662/548)	303–523	0.023(298-448K)	0.49%(298K)	[16]
Ho^3+^	Ca_3_La_6_Si_6_O_24_	^5^F_5_/(^5^F_4_,^5^S_2_)→^5^I_8_(658/546)	293–533	0.03(293-533K)	0.15%(293K)	[18]
Ho^3+^	CaMoO_4_	^5^F_3_/^3^K_8_ →^5^I_8_(460/489)	303–543	6.6 × 10^−3^(353K)	648.8/*T*^2^	[54]
Tm^3+^	K_3_Y(PO_4_)_2_	^3^F_2,3_/^3^H_4_→^3^H_6_(688/790)	293–553	0.304 × 10^−3^(553K)	1910.1/*T*^2^	[11]
Tm^3+^	Ba_3_La(PO_4_)_3_	^3^F_2,3_/^3^H_4_→^3^H_6_(690/792)	303–503	0.131 × 10^−3^(503K)	2.11%(303K)	[14]
Tm^3+^	KLuF_4_	^3^F_2,3_/^3^H_4_→^3^H_6_(690/795)	303–503	0.145 × 10^−3^(503K)	1249.85/T^2^	[56]
Er^3+^	Sr_3_Y(PO_4_)_3_	^2^H_11/2_/^4^S_3/2_→^4^I_15/2_(524/547)	298–573	5.07 × 10^−3^(523K)	1026.5/*T*^2^	This work
Er^3+^		^2^H_11/2_/^4^F_9/2_→^4^I_15/2_(524/655)		3.6 × 10^−3^ (298-573K)	1.11%(298K)	
Ho^3+^		^5^F_5_/(^5^F_4_,^5^S_2_)→^5^I_8_(656/543)		0.019(298-573K)	0.16%(298K)	
Ho^3+^		^5^I_4_/^5^F_5_→^5^I_8_(801/656)		0.46 × 10^−3^(573K)	0.35%(573K)	
Ho^3^		^5^I_4_/(^5^F_4_,^5^S_2_)→^5^I_8_(801/543)		9.39 × 10^−3^(573K)	0.42%(573K)	
Tm^3+^		^3^F_2,3_/^3^H_4_→^3^H_6_(695/795)		0.82 × 10^−3^(573K)	1547.7/*T*^2^	
Tm^3+^		^3^F_2,3_/^1^G_4_→^3^H_6_(695/476)		1.53 × 10^−3^(573K)	0.92%(298K)	
Tm^3+^		^3^F_2,3_→^3^H_6_/^1^G_4_→^3^F_4_(695/650)		12.74 × 10^−3^(573K)	1.52%(298K)	

The relative sensitivity (*S_R_*) determined with Equation (6) presents a continuous decrease with increasing temperature for both *I*_524_/*I*_547_ and *I*_524_/*I*_655_ FIRs (Figure 8(f)), but the use of *I*_524_/*I*_547_ produced a larger *S_R_* than *I*_524_/*I*_655_ on the whole. As presented in Table 1, the Sr_3_Y_0.88_(PO_4_)_3_:0.10Yb^3+^,0.02Er^3+^ phosphor has maximum *S_R_* values of ~1.16% (298 K) and 1.11% (298 K) for the *I*_524_/*I*_547_ and *I*_524_/*I*_655_ FIRs, respectively, which are slightly smaller than those of K_3_Y(PO_4_)_2_:Yb/Er [11] and NaYF_4_:Yb/Er [52] but are higher than those of Y_2_O_3_:Yb/Er [7], Ca_3_La_6_Si_6_O_24_:Yb/Er [18] and Ba_3_La(PO_4_)_3_:Yb/Er [14]. Judged from *S*_A_ and *S_R_* values, it can be concluded that Sr_3_Y_0.88_(PO_4_)_3_:0.10Yb^3+^,0.02Er^3+^ has a better performance of temperature sensing with thermally coupled ^2^H_11/2_/^4^S_3/2_ instead of non-thermally coupled ^2^H_11/2_/^4^F_9/2_ levels.

**Figure 9.**
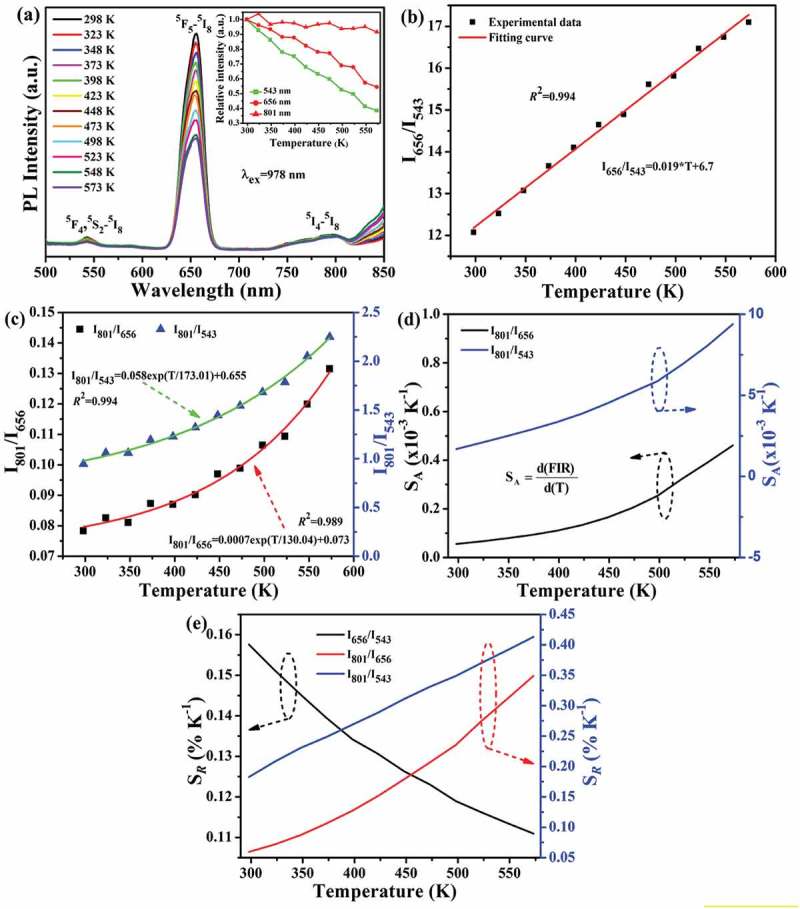
Temperature-dependent emission spectra under 1.00 W of 978 nm laser excitation (a), the dependences of *I*_656_/*I*_543_ (b) and *I*_801_/*I*_656_ and *I*_801_/*I*_543_ (c) FIRs on the absolute temperature, the absolute sensitivity (*S*_A_) of *I*_801_/*I*_656_ and *I*_801_/*I*_543_ FIRs (d), and the relative sensitivities (*S*_R_) of *I*_656_/*I*_543_, *I*_801_/*I*_656_ and *I*_801_/*I*_543_ FIRs (e) of the Sr_3_Y_0.88_(PO_4_)_3_:0.10Yb^3+^,0.02Ho^3+^ phosphor. The inset on part (a) shows relative intensities of the 543, 656 and 801 nm emissions as a function of the absolute temperature. Note the different scales of the vertical axes in parts (d) and (e).

Figure 9(a) presents the temperature-dependent UC spectra of Sr_3_Y_0.88_(PO_4_)_3_:0.10Yb^3+^,0.02Ho^3+^ under 1.00 W of 978 nm laser pumping, where it is seen that the green (543 nm; ^5^F_4_/^5^S_2_→^5^I_8_), red (656 nm; ^5^F_5_→^5^I_8_) and NIR (801 nm; ^5^I_4_ →^5^I_8_) bands continuously lose intensity towards a higher temperature but at different rates (Figure 9(a), the inset). As analyzed in Figure 9(b,c), *I*_656_/*I*_543_ FIR follows the linear equation of FIR(*I*_656_/*I*_543_) = 0.019*T+ 6.7, while *I*_801_/*I*_656_ and I_801_/I_543_ FIRs can be fitted with the single-exponential equations of FIR(*I*_801_/*I*_656_) = 0.0007exp(*T*/130.04)+0.073 and FIR(*I*_801_/*I*_543_) = 0.058exp(*T*/173.01)+0.655, respectively.

Though the *S*_A_ of *I*_801_/*I*_656_ and I_801_/I_543_ FIRs steadily increased from ~0.056 × 10^−3^ to 0.46 × 10^−3^ K^−1^ and from ~1.68 × 10^−3^ to 9.39 × 10^−3^ K^−1^ with the temperature increasing from 298 to 573 K (Figure 9(d)), respectively, the maximum values are yet lower than that of *I*_656_/*I*_543_ FIR (~0.019 K^−1^, Figure 9(b)). Accordingly, the maximum *S*_A_ is ~0.019 K^−1^ for the Sr_3_Y_0.88_(PO_4_)_3_:0.10Yb^3+^,0.02Ho^3+^ phosphor in the tested temperature range, which is lower than the values of K_3_Y(PO_4_)_2_:Yb/Ho [11], BaY_2_Si_3_O_10_:Yb/Ho [16] and Ca_3_La_6_Si_6_O_24_:Yb/Ho [18] but is higher than those of Y_2_O_3_:Yb/Ho [8] and CaMoO_4_:Yb/Ho [54] (Table 1). Figure 9(e) demonstrates the relative sensitivities derived with Equation (6). While the *S_R_* of *I*_656_/*I*_543_ FIR gradually decreased from ~0.16% to 0.12% K^−1^, those of *I*_801_/*I*_656_ and *I*_801_/*I*_543_ FIRs monotonically increased from ~0.059% to 0.35% K^−1^ and from ~0.19% to 0.42% K^−1^, respectively. The maximum *S_R_* (0.42% K^−1^) of this phosphor is higher than those of Yb^3+^/Ho^3+^ co-doped K_3_Y(PO_4_)_2_ [11] and Ca_3_La_6_Si_6_O_24_ [18], as compared in Table 1.

**Figure 10.**
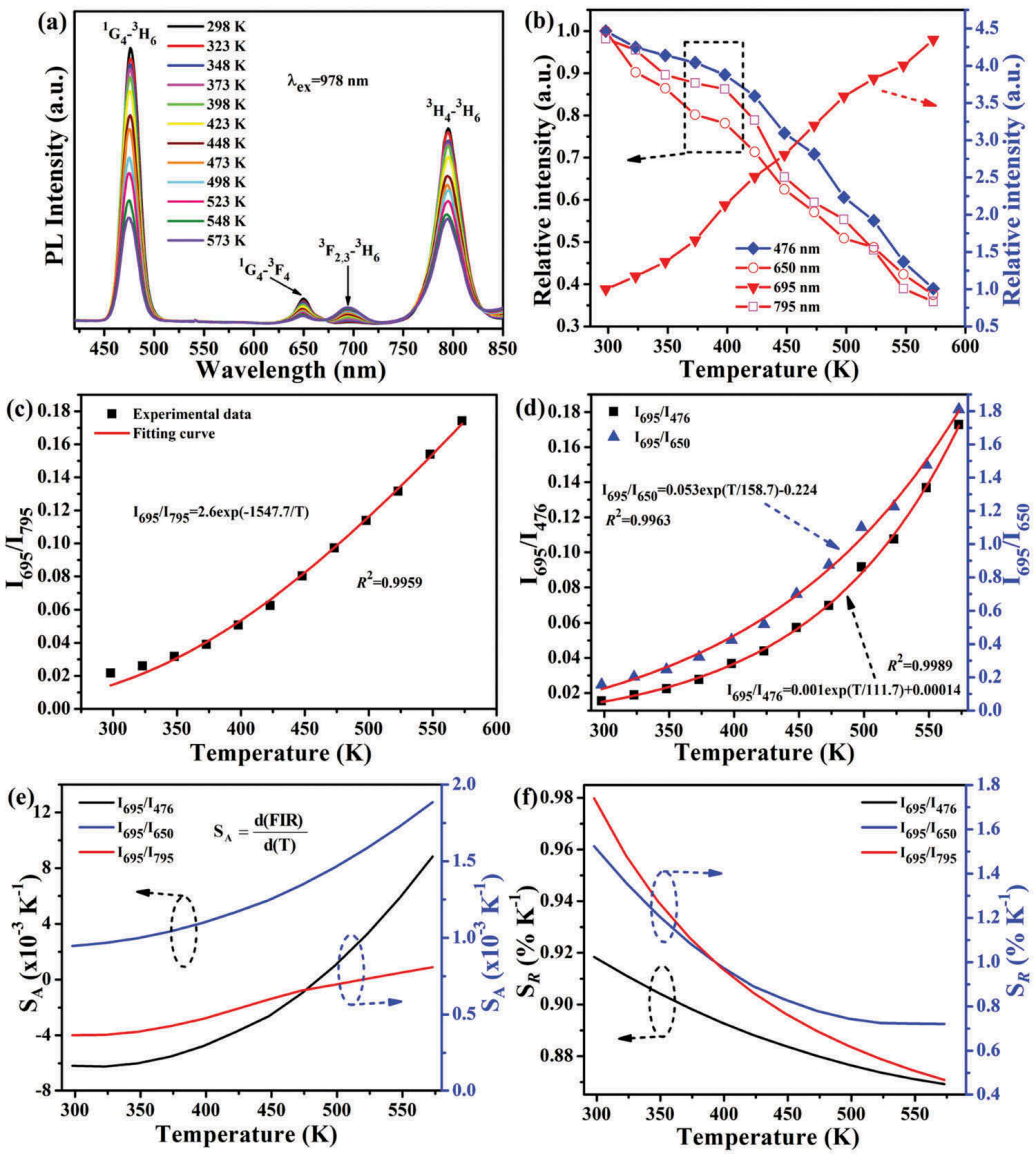
Temperature-dependent emission spectra under 1.00 W of 978 nm laser excitation (a), relative intensities of the 476, 650, 695 and 795 nm emissions as a function of the measurement temperature (b), the dependences of *I*_695_/*I*_795_ (c) and *I*_695_/*I*_476_ and *I*_695_/*I*_650_ (d) FIRs on the absolute temperature, and the absolute (e) and relative (f) sensitivities for the *I*_695_/*I*_476_, *I*_695_/*I*_650_ and I_695_/I_795_ FIRs of the Sr_3_Y_0.88_(PO_4_)_3_:0.10Yb^3+^,0.02Tm^3+^ phosphor. Note the different scales of the vertical axes in parts (b), (d), (e) and (f).

Figure 10(a) and (b), respectively, show temperature-dependent UC spectra and relative intensities of the emission bands for the Sr_3_Y_0.88_(PO_4_)_3_:0.10Yb^3+^,0.02Tm^3+^ phosphor under 1.00 W of 978 nm laser excitation. It was noticed that the intensities of ^1^G_4_→^3^H_6_ (476 nm), ^1^G_4_→^3^F_4_ (650 nm) and ^3^H_4_→^3^H_6_ (795 nm) emissions continuously decrease while that of ^3^F_2,3_→^3^H_6_ (695 nm) increases towards a higher temperature. Specifically, the 795 and 695 nm emissions were lowered by ~60% and enhanced by ~4.5 times at 573 K, respectively (Figure 10(b)), which is owing to the fact that ^3^F_2,3_ and ^3^H_4_ energy levels are thermally coupled and a higher temperature enhances population of the upper-lying ^3^F_2,3_ state [11]. Figure 10(c) plots the *I*_695_/*I*_795_ FIR as a function of the absolute temperature, where it was found that the data can be satisfactorily fitted with the single-exponential equation of FIR(*I*_695_/*I*_795_) = 2.6exp(−1547.7/*T*). The derived Δ*E* value (1075.7 cm^−1^) is significantly larger than that (713.42 cm^−1^) between the thermally coupled ^2^H_11/2_/^4^S_3/2_ levels of Er^3+^, which suggests that Yb^3+^/Tm^3+^ pair can be much better than Yb^3+^/Er^3+^ in Sr_3_Y(PO_4_)_3_ for temperature sensing. For the thermally coupled ^3^F_2,3_/^3^H_4_ energy levels, the error (δ) between estimated energy-gap (Δ*E*_e_~1075.7 cm^−1^) and experimentally measured energy-gap (Δ*E*_m_~1709.9 cm^−1^ from Figure 10(c)) can be calculated with the following expression [4,46,55] to be ~37.1%.

(7) $$\delta =\frac{\left|\Delta E_{e}-\Delta E_{m}\right|}{\Delta E_{m}}\times 100\%$$

The δ of this work is larger than the value (below 20%) [4] reported for Na(Lu,Gd)F_4_:Tm^3+^/Yb^3+^ but is smaller than those (above 40%) [4] for Tm^3+^/Yb^3+^ codoped NaNbO_3_ and Y_2_O_3_.

Non-thermally coupled energy levels were also analyzed to see their performance of temperature sensing, and Figure 10(d) shows the dependences of *I*_695_/*I*_476_ and *I*_695_/*I*_650_ FIRs on absolute temperature. It is encouraging to see that both the FIRs continuously increase with increasing temperature and the experimental data can be well fitted with the single-exponential equations of FIR(*I*_695_/*I*_476_) = 0.001exp(*T*/111.7)+0.00014 and FIR(*I*_695_/*I*_650_) = 0.053exp(*T*/158.7)-0.224. As analyzed in Figure 10(e,f), the *S*_A_ and *S*_R_ values gradually increase and decrease with increasing temperature, respectively. It is also seen that *I*_695_/*I*_650_ FIR has the largest *S*_A_ and *I*_695_/*I*_476_ FIR has the smallest *S*_R_ on the whole. It is also seen from the Figures that the ^1^G_4_ →^3^F_4_ (650 nm)/^3^F_2,3_→^3^H_6_ (695 nm) non-thermally coupled emissions have the largest *S*_A_ of ~12.74 × 10^−3^ K^−1^ at 573 K while the thermally coupled ^3^F_2,3_→^3^H_6_ (695 nm)/^3^H_4_→^3^H_6_ (795 nm) emissions have the largest *S_R_* value of ~1.74% K^−1^ at 298 K. As compared in Table 1, the Sr_3_Y_0.88_(PO_4_)_3_:0.10Yb^3+^,0.02Tm^3+^ phosphor has lower *S_R_* but significant higher *S*_A_ than K_3_Y(PO_4_)_2_:Yb/Tm [11], Ba_3_La(PO_4_)_3_:Yb/Tm [14] and KLuF_4_:Yb/Tm [56].

## 4. Conclusions

Eulytite-type Sr_3_Y_0.88_(PO_4_)_3_:0.10Yb^3+^,0.02Ln^3+^ phosphors (Ln = Ho, Er, Tm) were synthesized *via* gel-combustion, and their properties and mechanisms of UC luminescence as well as performances of optical temperature sensing with both thermally coupled and non-thermally coupled energy levels were systematically investigated. The main conclusions are summarized as follows:

(1) The Sr_3_Y(PO_4_)_3_:Yb^3+^/Ln^3+^ phosphors exhibit green (Ln = Er), orange-red (Ln = Ho) and blue (Ln = Tm) UC luminescence via two- and three-photon processes under 978 nm NIR laser excitation. The phosphors were analyzed to have the average decay times of ~52 ± 2, 260.6 ± 0.7 and 117 ± 1 μs for the 524 nm green, 657 nm red and 476 nm blue emissions of Er^3+^, Ho^3+^ and Tm^3+^, respectively.

(2) Sr_3_Y_0.88_(PO_4_)_3_:Yb^3+^/Er^3+^ exhibits a better performance of temperature sensing with the thermally coupled ^2^H_11/2_ and ^4^S_3/2_ energy levels, whose maximum absolute (*S*_A_) and relative (*S_R_*) sensitivities are ~5.07 × 10^−3^ K^−1^ at 523 K and ~1.16% at 298 K, respectively.

(3) Sr_3_Y_0.88_(PO_4_)_3_:Yb^3+^/Ho^3+^ shows maximum *S*_A_ and *S_R_* values of ~0.019 K^−1^ (298–573 K) and ~0.42% at 573 K for the non-thermally coupled energy pairs of ^5^F_5_/(^5^F_4_,^5^S_2_) and ^5^I_4_/^5^F_5_, respectively.

(4) Sr_3_Y_0.88_(PO_4_)_3_:Yb^3+^/Tm^3+^ has a maximum *S*_A_ of ~12.74 × 10^−3^ K^−1^ at 573 K for the non-thermally coupled ^3^F_2,3_→^3^H_6_/^1^G_4_→^3^F_4_ emissions and a maximum *S_R_* of ~1.74% K^−1^ at 298 K for the thermally coupled ^3^F_2,3_→^3^H_6_/^3^H_4_→^3^H_6_ emissions.
