# Supplementary material for: Upconversion luminescence and favorable temperature sensing performance of eulytite-type Sr3Y(PO4)3:Yb3+/Ln3+ phosphors (Ln=Ho, Er, Tm)
Source: Sci Technol Adv Mater. 2019 Aug 29;20(1):949–63. doi: 10.1080/14686996.2019.1659090 (PMC6764385; doi:10.1080/14686996.2019.1659090)
Supplement: Supplemental Material [file TSTA_A_1659090_SM6544.doc]

**Supporting Information**

**Upconversion luminescence and favorable temperature sensing performance of eulytite-type Sr_3_Y(PO_4_)_3_:Yb^3+^/Ln^3+^ phosphors (Ln=Ho, Er, Tm)**

**Weigang Liu,^a,b^ Xuejiao Wang,^c,d^ Qi Zhu,^a,b^ Xiaodong Li,^a,b^ Xudong Sun,^a,b^ and Ji-Guang Li^*d^**

^a^ Key Laboratory for Anisotropy and Texture of Materials (Ministry of Education), Northeastern University, Shenyang, Liaoning 110819, China

^b^ Institute of Ceramics and Powder Metallurgy, School of Materials Science and Engineering, Northeastern University, Shenyang, Liaoning 110819, China

^c^ College of New Energy, Bohai University, Jinzhou, Liaoning 121007, China

^d^ Research Center for Functional Materials, National Institute for Materials Science, Tsukuba, Ibaraki 305-0044, Japan

^*^Corresponding author

Dr. Ji-Guang Li

National Institute for Materials Science

Tsukuba, Japan

Tel: +81-29-860-4394

E-mail: [li.jiguang@nims.go.jp](mailto:li.jiguang@nims.go.jp)

**Table S1.** The position (*x*, *y*, *z*) and site occupancy factor (SOF) of atoms derived via Rietveld refinement for Sr_3_Y_0.88_(PO_4_)_3_:0.10Yb^3+^,0.02Er^3+^.

| Atom | Valence | Wyckoff symbol | *x* | *y* | *z* | SOF |
| --- | --- | --- | --- | --- | --- | --- |
| Sr | +2 | 16c | 0.0646 (1) | 0.0646 (1) | 0.0646 (1) | 0.7463 |
| Y | +3 | 16c | 0.0646 (1) | 0.0646 (1) | 0.0646 (1) | 0.2267 |
| Yb | +3 | 16c | 0.0646 (1) | 0.0646 (1) | 0.0646 (1) | 0.0233 |
| Er | +3 | 16c | 0.0646 (1) | 0.0646 (1) | 0.0646 (1) | 0.0037 |
| P | +5 | 12a | 0.375 | 0.000 | 0.25 | 1 |
| O1 | -2 | 48e | 0.1223(2) | 0.1217(2) | 0.3417(2) | 0.3442 |
| O2 | -2 | 48e | 0.9784(2) | 0.0820(2) | 0.2933(2) | 0.4644 |
| O3 | -2 | 48e | 0.0496(1) | 0.2912(1) | 0.4859(1) | 0.1914 |


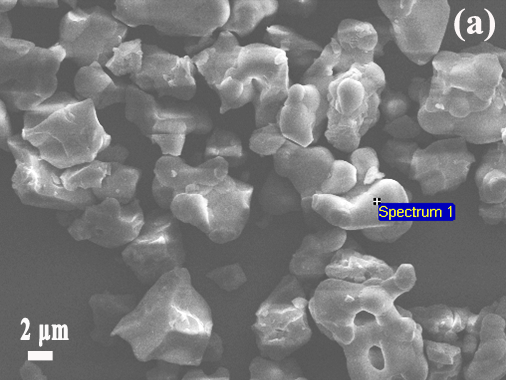

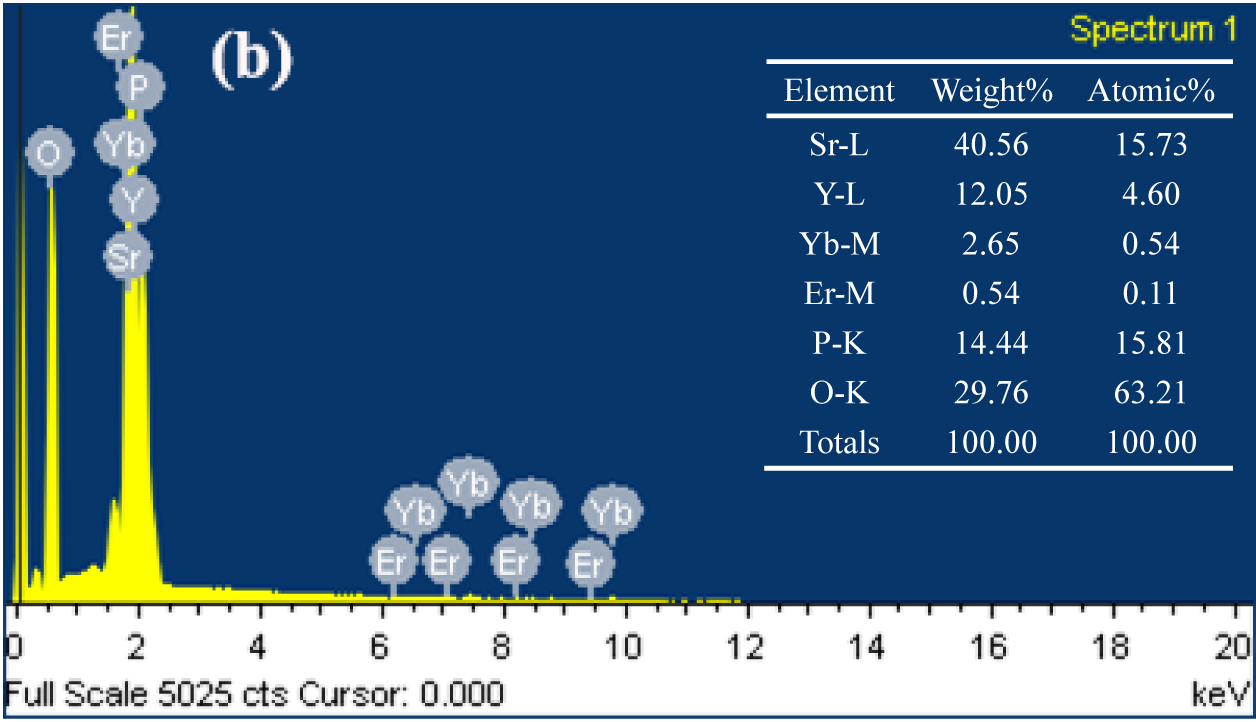


**Figure S1.** FE-SEM morphology (a) and the results of EDS composition analysis (b) of the Sr_3_Y_0.88_(PO_4_)_3_:0.10Yb^3+^,0.02Er^3+^ sample.


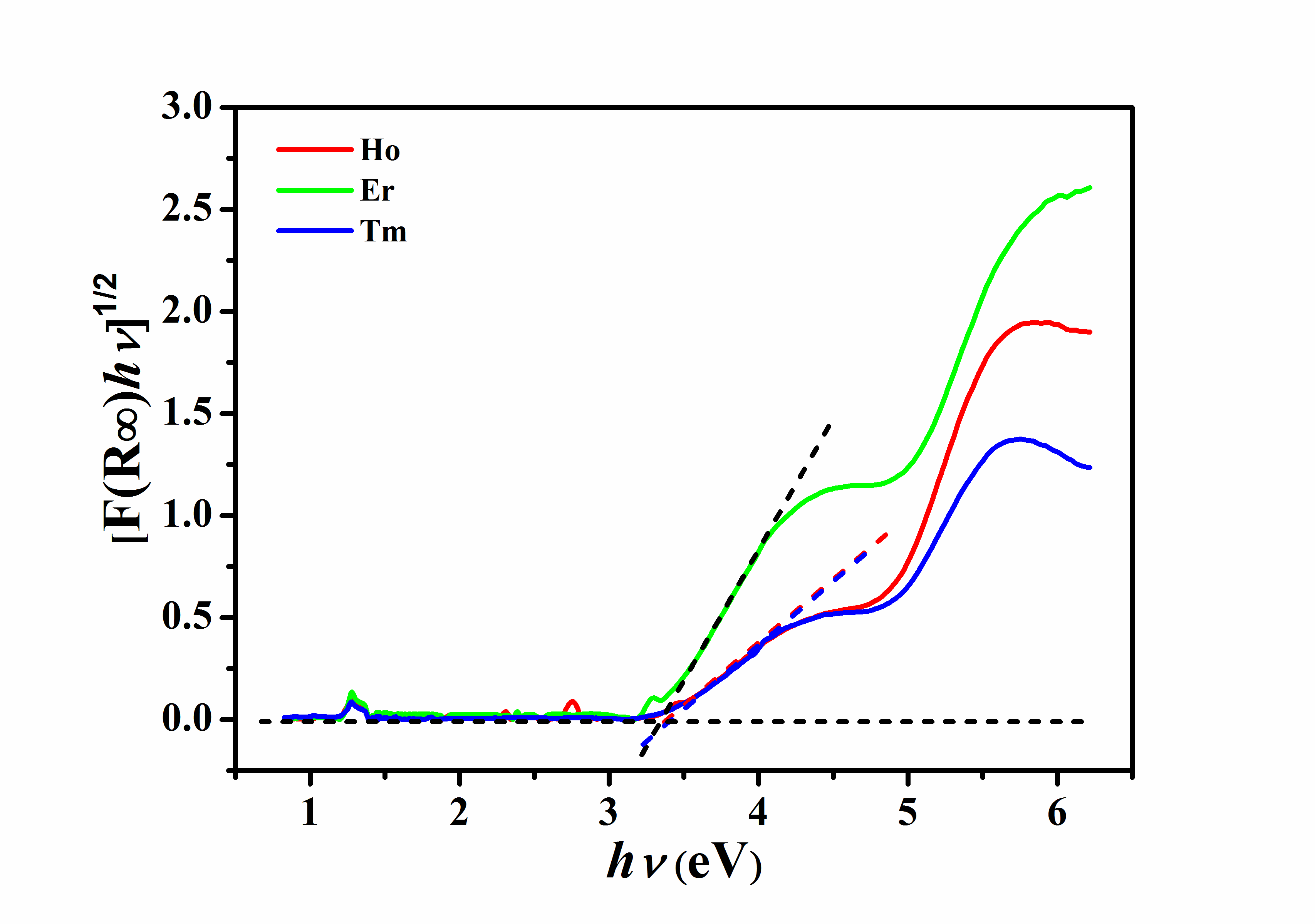


**Figure S2.** The determination of bandgap energy for Sr_3_Y_0.88_ (PO_4_)_3_:0.10Yb^3+^,0.02Ln^3+^.

**Table S2.** CIE chromaticity coordinates (*x*, *y*) for the upconversion luminescence of Sr_3_Y_0.88_(PO_4_)_3_:0.10Yb^3+^,0.02Ln^3+^ (Ln=Er, Ho and Tm) under varying excitation power.

| Excitation power (W) | Er | Ho | Tm |
| --- | --- | --- | --- |
| 1.00 | (0.3288,0.5102) | (0.5503,0.4318) | (0.1764,0.1781) |
| 1.25 | (0.3235,0.5388) | (0.5678,0.4167) | (0.1622,0.1602) |
| 1.50 | (0.315,0.5574) | (0.5804,0.4055) | (0.1512,0.1466) |
| 1.75 | (0.3025,0.5748) | (0.5964,0.3912) | (0.1469,0.1395) |
| 2.00 | (0.2956,0.5848) | (0.6015,0.3868) | (0.1412,0.1334) |
| 2.25 | (0.2892,0.5953) | (0.6070,0.3818) | (0.1385,0.1291) |
| 2.50 | (0.2795,0.611) | (0.6171,0.373) | (0.1362,0.1256) |
| 2.75 | (0.2716,0.6206) | (0.6226,0.368) | (0.1336,0.1209) |
| 3.00 | (0.2671,0.6276) | (0.6235,0.3676) | (0.1323,0.1194) |


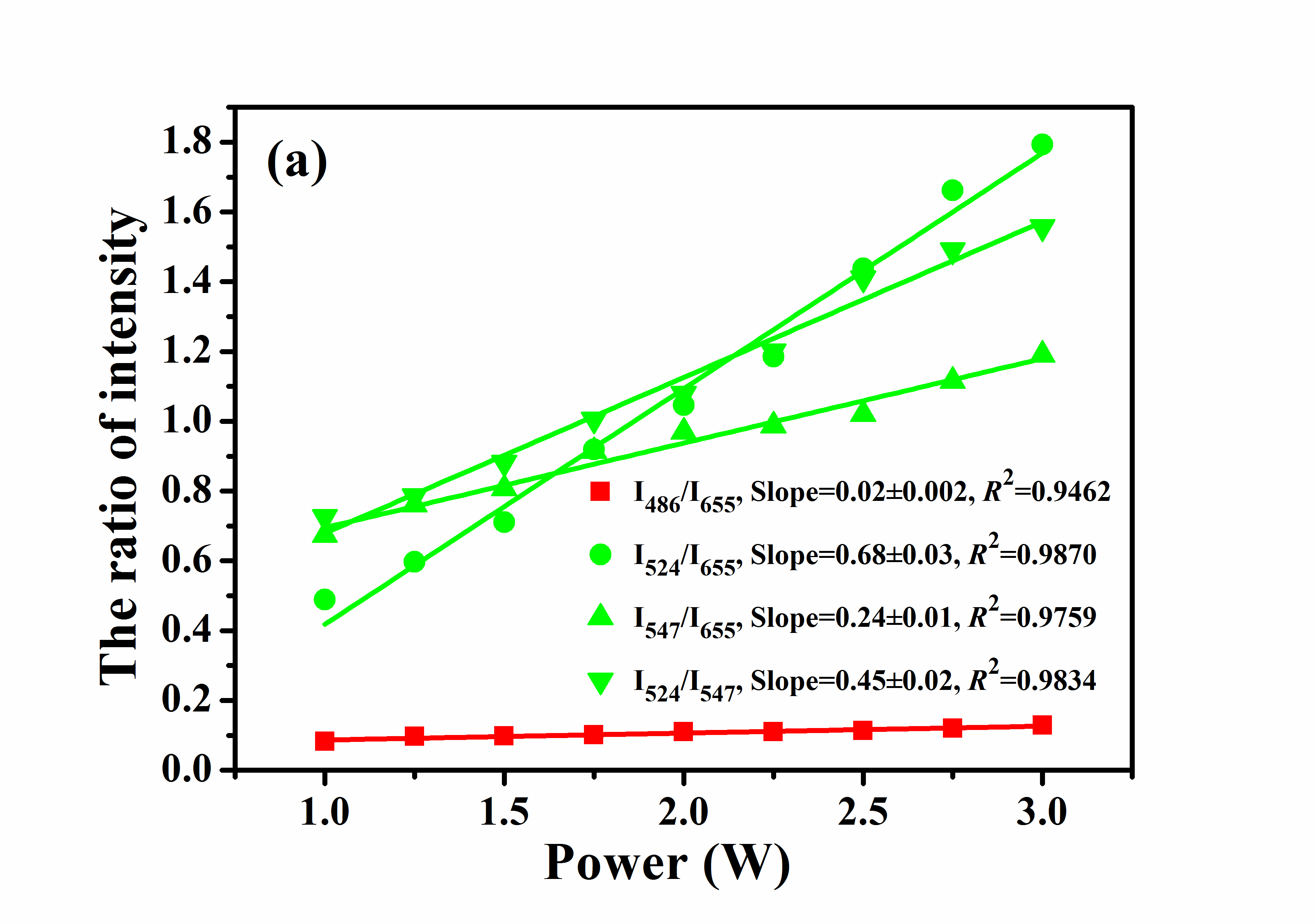

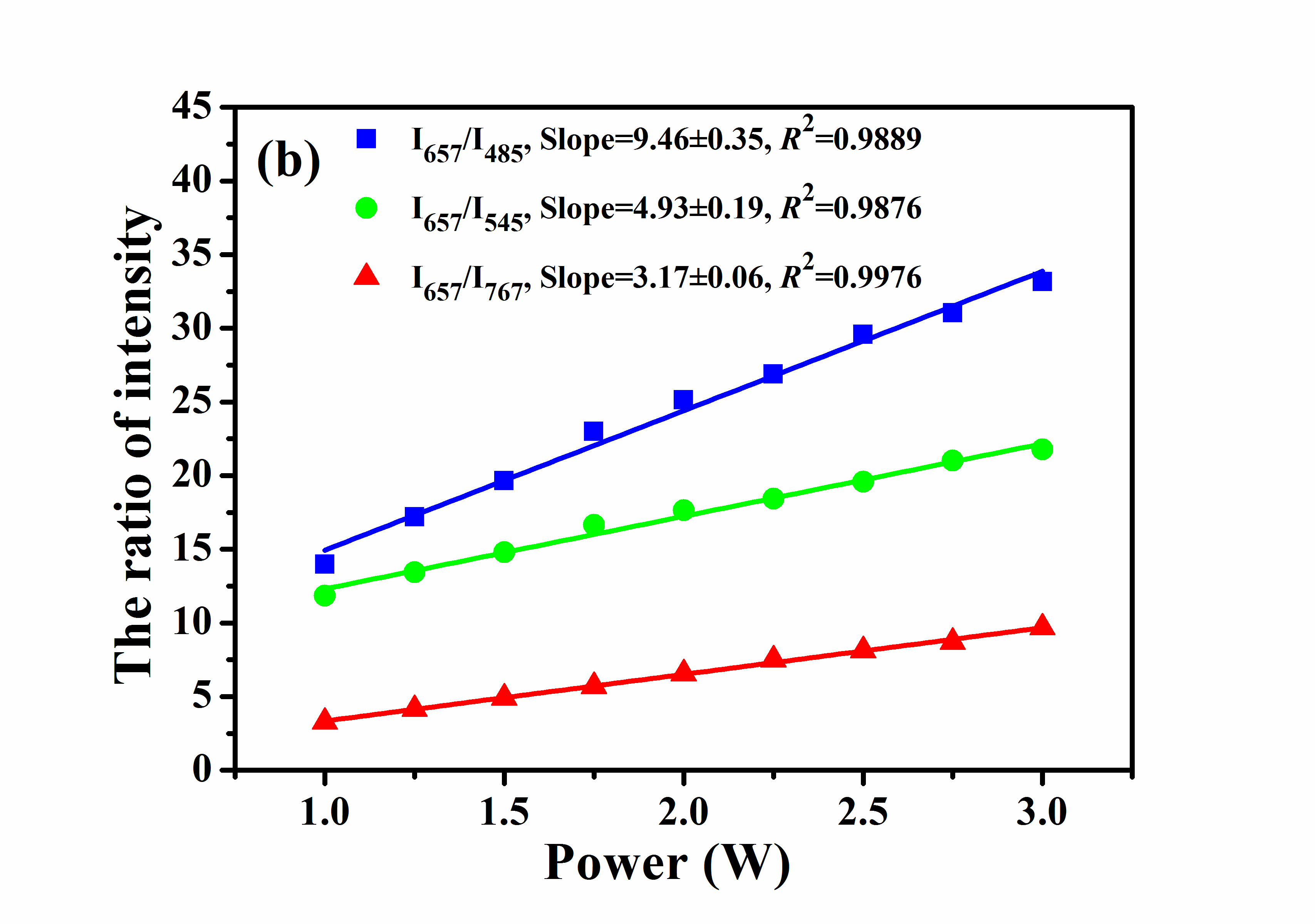

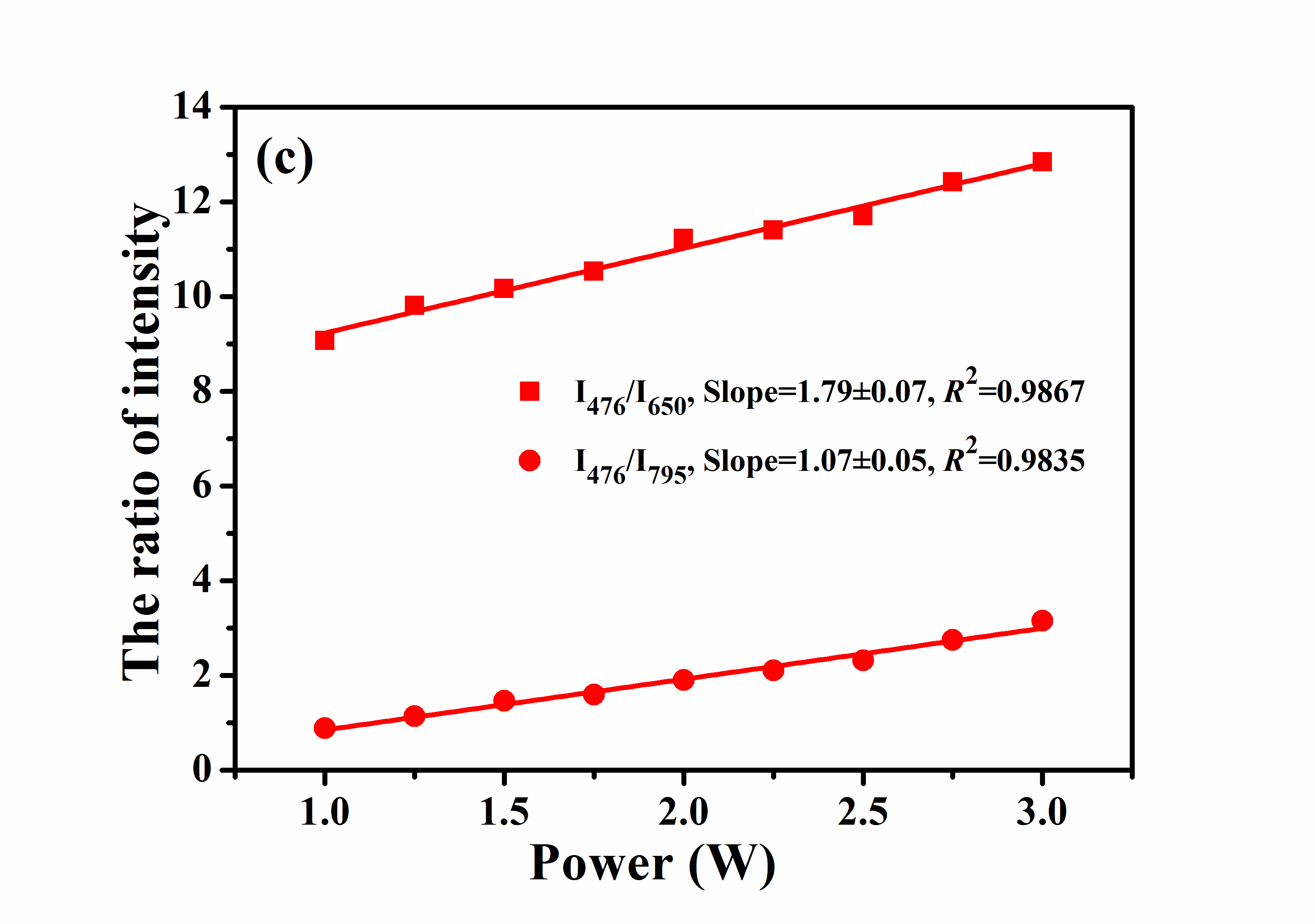


**Figure S3.** Intensity ratio of the characteristic UC emissions of Sr_3_Y_0.88_(PO_4_)_3_:0.10Yb^3+^,0.02Ln^3+^ under varying excitation power, where Ln=Er (a), Ho (b) and Tm (c). The solid lines are the results of linear fitting.

**Table S3.** The results of fluorescence lifetime analysis for the Sr_3_Y_0.88_(PO_4_)_3_:0.10Yb^3+^,0.02Ln^3+^ phosphors (Ln=Er, Ho and Tm).

| Ln | Er | Ho | Tm |
| --- | --- | --- | --- |
| λ_em_ (nm) | 524 | 657 | 476 |
| Lifetime τ_1_ (μs) | 21±2 | 29.6±0.6 | 41.7±0.6 |
| Weight of τ_1_ (%) | 19.53 | 16.44 | 46.07 |
| Lifetime τ_2_ (μs) | 59.8±1.3 | 306.1±1.1 | 181.4±1.5 |
| Weight of τ_2_ (%) | 80.47 | 83.56 | 53.93 |
| Chi-square | 1.03 | 1.73 | 1.16 |
| Lifetime τ* (μs) | 52±2 | 260.6±0.7 | 117±1 |
